# Necrosis-like cell death modes in heart failure: the influence of aetiology and the effects of RIP3 inhibition

**DOI:** 10.1007/s00395-025-01101-4

**Published:** 2025-03-15

**Authors:** Izabela Jarabicová, Csaba Horváth, Jaroslav Hrdlička, Almos Boroš, Veronika Olejníčková, Eva Zábrodská, Soňa Štemberková Hubáčková, Hana Mauer Šutovská, Ľuboš Molčan, Libor Kopkan, Martin Chudý, Branislav Kura, Barbora Kaločayová, Eva Goncalvesová, Jan Neckář, Michal Zeman, František Kolář, Adriana Adameová

**Affiliations:** 1 Faculty of Pharmacy, Department of Pharmacology and Toxicology, Comenius University, Odbojárov 10, 832 32 Bratislava, Slovak Republic; 2 Institute of Physiology, Czech Academy of Sciences, Prague, Czech Republic; 3 First Faculty of Medicine, Institute of Anatomy, Charles University, Prague, Czech Republic; 4 Institute for Clinical and Experimental Medicine, Prague, Czech Republic; 5 Institute of Biotechnology, Czech Academy of Sciences, Prague, Czech Republic; 6 Faculty of Natural Sciences, Department of Animal Physiology and Ethology, Comenius University, Bratislava, Slovak Republic; 7 Faculty of Medicine, Department of Cardiology, Comenius University and National Cardiovascular Institute, Bratislava, Slovak Republic; 8 Centre of Experimental Medicine, Institute for Heart Research, Slovak Academy of Sciences, Bratislava, Slovak Republic

**Keywords:** Heart failure, Receptor-interacting protein kinase 3, Inflammation, High mobility group box 1, Necroptosis, Pyroptosis

## Abstract

Since cell dying in heart failure (HF) may vary based on the aetiology, we examined the main forms of regulated necrosis, such as necroptosis and pyroptosis, in the hearts damaged due to myocardial infarction (MI) or pressure overload. We also investigated the effects of a drug inhibiting RIP3, a proposed convergent point for both these necrosis-like cell death modes. In rat hearts, left ventricular function, remodelling, pro-cell death, and pro-inflammatory events were investigated, and the pharmacodynamic action of RIP3 inhibitor (GSK'872) was assessed. Regardless of the HF aetiology, the heart cells were dying due to necroptosis, albeit the upstream signals may be different. Pyroptosis was observed only in post-MI HF. The dysregulated miRNAs in post-MI hearts were accompanied by higher levels of a predicted target, HMGB1, its receptors (TLRs), as well as the exacerbation of inflammation likely originating from macrophages. The RIP3 inhibitor suppressed necroptosis, unlike pyroptosis, normalised the dysregulated miRNAs and tended to decrease collagen content and affect macrophage infiltration without affecting cardiac function or structure. The drug also mitigated the local heart inflammation and normalised the higher circulating HMGB1 in rats with post-MI HF. Elevated serum levels of HMGB1 were also detected in HF patients and positively correlated with C-reactive protein, highlighting pro-inflammatory axis. In conclusion, in MI-, but not pressure overload-induced HF, both necroptosis and pyroptosis operate and might underlie HF pathogenesis. The RIP3-targeting pharmacological intervention might protect the heart by preventing pro-death and pro-inflammatory mechanisms, however, additional strategies targeting multiple pro-death pathways may exhibit greater cardioprotection.

**Supplementary Information:**

The online version contains supplementary material available at 10.1007/s00395-025-01101-4.

## Introduction

Heart failure (HF) is one of the leading causes of death and disability in both high- and low-income countries. HF, in particular with reduced ejection fraction (HFrEF), is associated with various pathological cellular and molecular changes in the heart resulting in impaired cardiac function and adverse remodelling [31]. Besides cardiac causes, extracardiac mechanisms (e.g. systemic inflammation) are presumable pathomechanisms of HF that underlie the progression of the disease and the severity of episodes for decompensation of ventricular function [2, 32]. Current guideline-directed medical therapy for HF mainly aims to target the local, cardiac pathomechanisms [14]. Although such therapy significantly improves cardiac function and survival in HF patients, the prevalence of this syndrome is on the rise and has become a major economic burden to individuals and health systems [33]. Furthermore, in some patients, the efficacy of the therapy and the prognosis of HF are very poor and thus they die within a few years [14, 33].

Necrosis-like cell death, such as necroptosis and pyroptosis, have been identified in settings of myocardial ischaemia/reperfusion injury and failing hearts of various aetiology [1, 15, 16, 20, 24]. The signalling of necroptosis is mediated by receptor-interacting protein kinase 3 (RIP3) which activates the main cytotoxic protein—mixed lineage kinase domain-like pseudokinase (MLKL) to form homo/hetero-oligomers [11, 26]. Translocation of these large protein structures to the plasma membrane resulting in cell oncosis and rupture leads to the release of the intracellular fluid containing damage-associated molecular patterns (DAMPs), such as high mobility group box 1 (HMGB1), to further promote the pro-inflammatory environment [43]. The RIP3–MLKL signalling can be activated by RIP1 which is known as canonical necroptosis. On the other hand, non-canonical necroptosis is promoted by adaptor proteins, such as Toll-interleukin-1 receptor domain-containing adapter-inducing interferon (TRIF) or DNA-dependent activator of interferon regulatory factors/Z-DNA binding protein 1 (DAI/ZBP1) to directly interact with RIP3 [21, 41]. In addition to these two major necroptotic signallings, several other alternative necroptosis pathways with a key role for RIP3 have been proposed to mediate cardiac damage due to this type of cell death [17].

Pyroptosis is initiated by the formation of the molecular platform called inflammasome consisting of a nucleotide-binding domain, leucine-rich–containing family, pyrin domain–containing-3 (NLRP3), apoptosis-associated speck-like protein (ASC) and procaspase-1 [23]. Within this complex, procaspase-1 is autoproteolytically activated to convert pro-interleukin-1β (proIL-1β) and proIL-18 into their active forms which are extracellularly released thereby amplifying the inflammatory response. Active caspase-1 also cleaves gasdermin D (GSDMD) to generate its N-terminal fragments which oligomerize and execute pyroptosis by the formation of the membrane pores [4, 5].

As these two cell death modes lead to the significant loss of cardiac cells, including the cells of the working myocardium, they might, at least in part, underlie the pathogenesis of HF [25, 29]. Inflammation is a common feature of these pro-death events. Local inflammation occurs partly as an acute result of the DAMP-producing plasma membrane disruption due to the MLKL and N-GSDMD forming pores whilst chronic inflammatory response within the heart can be promoted as a consequence of the maladaptive activation of the immune system [7, 53]. Although necroptosis and pyroptosis can operate separately, there is growing evidence indicating an interlink between their signalling pathways with an important role of RIP3 [9, 23]. Thus, these cell death modes, in particular when co-existing, might be a vicious circle producing inflammation thereby exacerbating the initial damage of the heart and promoting progression in the cardiac dysfunction and adverse remodelling.

In the context of this theory, we performed a comprehensive analysis of the signallings of both non-canonical and alternative necroptosis with respect to its interlink to pyroptotic cell death with a crucial role of RIP3 in rat hearts failing due to different reasons. As a follow-up to this analysis characterising a mechanistic link between the two pro-death, necrosis-like events, the pharmacological inhibition of RIP3 by GSK'872 was studied in post-myocardial infarction (MI) cardiac damage induced by left anterior descending artery (LAD) ligation. We tested for the first time a hypothesis that RIP3 inhibition can affect the pathomechanisms of post-MI HF by retarding both these cell death modes. Circulating molecules being relevant to the pro-death, pro-inflammatory events, along with the effect of the used pharmacological intervention were also investigated. To highlight the translational approach of the study, we also provided a screening in the samples of patients with HFrEF.

## Materials and methods

### Experimental models and study design

All procedures conform to the Guide for the Care and Use of Laboratory Animals published by the US National Institutes of Health (revised 2011) [10]. The experiments were approved by the Committee for Animal Care and Use at the Institute of Physiology of the Czech Academy of Sciences (Prague, Czech Republic) # 39/2021, Institute of Clinical and Experimental Medicine (IKEM, Prague, Czech Republic) # 27/2019 and conducted following the recommendations of ARRIVE guidelines, Guide for the Care and Use of Laboratory Animals and EU Directive 2010/63/EU for animal experiments. All efforts were made to minimise the number of rats used in this study.

All animals were housed under controlled laboratory conditions in a room with a constant temperature of 22 °C, humidity of 55–75% and a regular 12 h:12-h light/dark regime and were fed with a standard pellet diet and tap water ad libitum. The animals included in this study were randomly distributed to the investigated, previously confirmed models of HF due to either post-MI cardiac damage induced by temporary LAD ligation [24] or pressure overload-induced cardiac damage either by abdominal aortic constriction (AAC) [30] or using a transgenic (mRen-2)27 rat (TGR) model [37]. The respective models are further described below. At the end of each experiment, the animals were sacrificed by intraperitoneal injection of pentobarbital at the dose of 800 mg/kg and the hearts were rapidly excised and washed in ice-cold PBS. The left ventricles (LVs) were frozen in liquid nitrogen and kept at -80 °C until further analyses.

#### Post-myocardial infarction heart failure induced by LAD ligation

After the incubation period, adult male Wistar rats (250–300 g, Institute of Physiology of the Czech Academy of Sciences, Prague, Czech Republic) were randomly assigned into the following groups: untreated sham‐operated animals (Sham, *n* = 5), animals subjected to LAD ligation with subsequent development of HF being (*n* = 10) or not (*n* = 14) treated with GSK'872 for 7 days (5 mg/kg/d, ip). The dose of the drug was chosen according to the previously published studies employing the same route of drug administration [8, 51]. In anaesthetized open‐chest animals (sodium pentobarbital, 60 mg/kg ip), ischaemia was induced by the ligation of LAD 1‐2 mm distal to the left atrial appendage for 1 h, then the ligation was released resulting in ischaemia and reperfusion injury. Sham‐operated rats underwent chest surgery without the occlusion. The RIP3 inhibitor was applied daily for 7 days whilst the first dose of the drug was administrated immediately after LAD ligation release and then every 24 h. After chest closure, all spontaneously breathing animals recovering from anaesthesia were housed in separate cages with given analgesia (ibuprofen, 20 mg/d po). The mortality of post-MI rats was 50% (7/14) in untreated and 20% (2/10) in GSK'872-treated animals. Seven days after ischaemia and reperfusion injury, the rats were sacrificed as described above. Blood samples were taken from the right ventricular cavity using a needle mounted on an EDTA-coated S-Monovette® 7.5 ml K3E tube (Sarstedt, Germany) and were afterwards centrifuged (10 min, 5371 rpm) to get the plasma for further analyses. After the heart dissection, the whole free LV wall of sham‐operated animals was harvested whilst in the HF group, the LV was dissected into the infarcted area (HFi) and the surrounding non‐infarcted tissue (HFni). A schematic illustration of the experimental protocol of post-MI HF is shown in Fig. 4.

#### Pressure overload-induced cardiac damage

Pressure overload in newborn male Wistar rats (Institute of Physiology of the Czech Academy of Sciences, Prague, Czech Republic) was induced at the age of 2 days by AAC (*n* = 7) as described earlier [30]. Briefly, under ether anaesthesia (1.9%), the abdominal cavity was opened by an incision from the left dorsolateral side and the aorta was exposed in the subdiaphragmatic suprarenal region. A silk suture was tied around the aorta using a hypodermic needle of 0.25 mm in outer diameter as a template. The incision was sutured and smeared with 1% iodocollodium. Analgesia (Metamizole, Two hundred mg/kg in the drinking water of the nursing mother) was given for 3 days after the surgery. In sham-operated animals (*n* = 6), the aorta was exposed but not constricted. The animals were sacrificed at the age of 90 days. In another model of pressure overload, induced by Ren-2 overexpression, adult male TGR rats (*n* = 6) and HanSD rats (*n* = 3) as transgene-negative controls at the age of 14–16 weeks (250–300 g, IKEM, Prague, Czech Republic) were used.

### Echocardiography

The geometry and function of the LV were assessed by echocardiography using GE Vivid 7 Dimension (GE Vingmed Ultrasound, Horten, Norway) with a 12 MHz linear matrix probe M12L. Animals were anaesthetized with 2% isoflurane (Forane, Abbott Laboratories, United Kingdom) mixed with room air, placed on a heating pad and their rectal temperature was maintained between 35.5 and 37.5 °C. Basic 2-D and M modes in both the long and short axes were recorded. The following parameters of LV geometry were assessed: end-diastolic and end-systolic LV cavity diameter (LVDd, LVDs), and posterior and anterior wall thickness in diastole (PWTd, AWTd). Fractional shortening (FS) and ejection fraction (EF) were derived as follows: FS = 100 × [(LVDd-LVDs)/LVDd]; EF = 100 × [(LVDd3 + LVDs3)/LVDd3].

In the post-MI HF model, echocardiography was performed 4 days prior to the surgery (due to the potential cardioprotective effects of isoflurane) and 7 days after the surgery. In the AAC and TGR models, echocardiography was performed at the end of the experiment.

### Immunoblotting

As described previously, immunoblot analysis was performed on whole-cell lysates from the LVs [20]. Briefly, following a post-electrophoretic transfer of proteins (under reducing or non-reducing conditions [38, 39]), the membranes were incubated with the primary antibodies indicated in Table S1. Subsequently, the membranes were incubated with the following secondary HRP-conjugated antibodies: donkey anti‐rabbit IgG (711‐035‐152, Jackson Immunoresearch), donkey anti‐rat IgG (112‐035‐175, Jackson Immunoresearch, USA) and donkey anti‐mouse IgG (115‐035‐174, Jackson Immunoresearch, USA). Signals were detected using enhanced chemiluminescence (Crescendo Luminata, Merck Millipore, USA) and captured by a chemiluminescence imaging system (myECL imager, Thermo Scientific, USA). Total protein staining of membranes with Ponceau S assessed by scanning densitometry was used as the loading control in total tissue lysates [35]. Relative expression of protein bands of interest was calculated by normalising the intensity of a protein band with its whole lane protein staining intensity.

### Immunohistochemistry

Tissues were snap-frozen immediately after resection from sacrificed animals. They were fixed overnight with 2% formaldehyde, washed extensively with PBS, incubated in DMSO (Sigma, USA) for 1 h at room temperature (RT) and subsequently in 30% sucrose (Sigma, USA) overnight at 4 °C. After fixation, tissues were incubated in increasing concentration of OCT mounting medium (20%, 50%, 70% diluted in 30% sucrose) and frozen in 100% OCT (Sigma, USA). Cryo-Sects. (5 µm) were permeabilized by combination of DMSO (15 min at RT) and 0.5% saponin (15 min at RT; Thermo Fisher, USA) in two consecutive steps. After washing with PBS, tissues were incubated in 10% FBS (diluted in PBS) for 30 min to block unspecific signals and with diluted primary antibody against F4/80 overnight at 4 °C (#70076; Cell Signaling Technology, USA, 1:100 in PBS). Samples were further washed with PBS and incubated for 1 h at RT with a secondary antibody—goat anti-rabbit Alexa Fluor 488 IgG (A11006, Thermo Fisher, USA). To counterstain nuclei, coverslips were mounted in Mowiol containing 4',6-diamidino-2-phenylindole (DAPI; Sigma, USA) and viewed in Olympus CX43 (Leica, Germany).

### TUNEL

Tissues were mounted into cryo-blocks and sectioned as described above. Cryo-Sects.  Five μm (prepared as described above) were permeabilized by combination of DMSO (15 min in RT; Sigma, USA) and 0.5% saponin (15 min in RT; Thermo Fisher, USA) in two consecutive steps. Cell death was detected using In Situ Cell Death Detection Kit, Fluorescein (11,684,795,910, Roche, Switzerland) according to the manufacturer’s instructions. DAPI (1 μg/mL) staining was used to visualise cell nuclei.

### Histology

For paraffin embedding, tissue was fixed overnight in 2% PFA, placed in 70% EtOH, and processed using the Leica ASP6025 autoprocessor. The programme was set to 12-h standard dehydration and paraffin saturation protocol. After processing, tissue samples were embedded into formalin-fixed paraffin blocks upside down by cutting their surface and orienting the blocks transversally. Standard PicoSirius Red staining, dehydration, and placing the samples on coverslips were accomplished using the Leica ST5020 automated staining instrument in combination with the Leica CV5030 cover-slipper.

### Human serum samples

The study using patients’ samples of the serum conforms to the Declaration of Helsinki and was approved by the ethics committee of the National Institute for Cardiovascular Diseases in Bratislava, Slovakia. The written informed consent was obtained from all participants of the study.

The patients’ serum samples were collected during hospitalisation for recently diagnosed HFrEF in the Department of Heart Failure and Heart Transplantation of the National Institute for Cardiovascular Diseases, Bratislava, Slovakia, as described previously [19].

### ELISA

The circulating HMGB1 levels were measured by commercially available enzyme-linked immunosorbent assay (ELISA) with the specificity for rats and humans (LSBio, Washington, USA) following the manufacturer´s instructions.

### Analysis of miRNA

The miRCURY LNA miRNA PCR Assay (QIAGEN, Hilden, Germany) was used for screening of any alterations in microRNAs (miRNAs) in the rat post-MI failing hearts according to the manufacturer´s instructions. At first, a total RNA was extracted from LVs by miRNeasy Tissue/Cells Advanced Mini Kit (QIAGEN, Hilden, Germany) according to the manufacturer´s protocol. Then both the concentration and purity of the total RNA were verified spectrophotometrically by Nanodrop ND-1000 (Agilent Technologies, USA) and reverse transcribed to cDNA by miRCURY LNA RT Kit (QIAGEN, Hilden, Germany). The qPCR reaction itself was performed by miRCURY LNA SYBR Green PCR Kit (QIAGEN, Hilden, Germany) on CFX96 Touch (BioRad, USA) on the thermal programme presented in Table S2. The final expression of miRNAs was calculated by the GenGlobe web tool (https://geneglobe.qiagen.com/, QIAGEN, Hilden, Germany). After that, quantitative real time polymerase chain reaction (RT-qPCR) was used to verify the data on the pre-selected miRNA profile and to find out the ability of the RIP3 inhibitor to reverse them. Total RNA was isolated by mirVana™ miRNA Isolation Kit (Thermo Fisher Scientific, Waltham, MA, USA) and then measured for purity with spectrophotometer Nanodrop ND-1000 (Agilent Technologies, USA) at 260 nm and 280 nm. Ten ng of the total RNA was reverse transcribed to cDNA with TaqMan™ Advanced miRNA cDNA Synthesis Kit (Thermo Fisher Scientific, Waltham, MA, USA), according to the manufacturer’s instructions. cDNA was amplified using the TaqMan™ Fast Advanced Master Mix for qPCR (Thermo Fisher Scientific, Waltham, MA, USA) with specific miRNA assays for miRNA-140-5p (Assay ID rno480932_mir) and miRNA-142-3p (Assay ID rno480934_mir) (Thermo Fisher Scientific, Waltham, MA, USA). All measured data were normalised to miRNA-103a-3p (Assay ID 478253_mir) (Thermo Fisher Scientific, Waltham, MA, USA). RT-qPCR analyses were performed on CFX96 Touch (BioRad, USA) on the thermal programme presented in Table S3. All obtained data from RT-qPCR were calculated by the 2^–∆∆Ct^ method. The mature sequences of the measured miRNAs are presented in Table S4.

### RT-qPCR

Small pieces of tissue (1–2 mm^3^) were placed into 500 µL of RNAzol (BioRad, Hercules, CA, USA), homogenised (2 × 30 s, 5 600 rpm) using the Precellys 24 homogenizer (Bertin Instruments, Montigny-le-Bretonneux, France) and cleared by centrifugation. Total RNA was isolated according to the manufacturer´s protocol. First strand cDNA was synthesised from 200 ng of total RNA with random hexamer primers using Revert Aid First strand cDNA Synthesis Kit (Thermo Fisher, USA). RT-qPCR was performed using the C1000™ Thermal Cycler (BioRad, USA) with 5 × HOT FIREPol® Evagreen® qPCR Supermix (Solis Biodyne, Tartu, Estonia). Relative quantity of cDNA was estimated by the ΔΔCT method, data were normalised to housekeeping gene β-actin. The primers (Table S5) were purchased from Sigma, USA.

### LDH assay

The levels of lactate dehydrogenase (LDH) were measured in the rat plasma after the end of the experiment. A commercially available colorimetric LDH Assay Kit (Abcam, UK) following the manufacturer’s instructions was used.

### Statistical analysis

The data are presented as mean ± standard error of mean (SEM). An unpaired t test or Mann–Whitney test (based on the normality of the data) was used to compare the two groups. For more than two groups, one-way analysis of variance (ANOVA) with Tukey’s post hoc test, two-way ANOVA with Tukey’s post hoc test or Kruskal–Wallis’s test with post hoc Dunn’s test was used to evaluate the differences, as appropriate. To determine the relationship between the circulating HMGB1 and selected parameters, Pearson correlation was applied. *p* < 0.05 was considered as significant. All statistical analyses were performed using GraphPad Prism, version 10.00 (GraphPad Software, USA).

## Results

### Functional and molecular analyses of failing hearts of different aetiologies

#### Heart function and myocardial remodelling

**Fig. 1:**
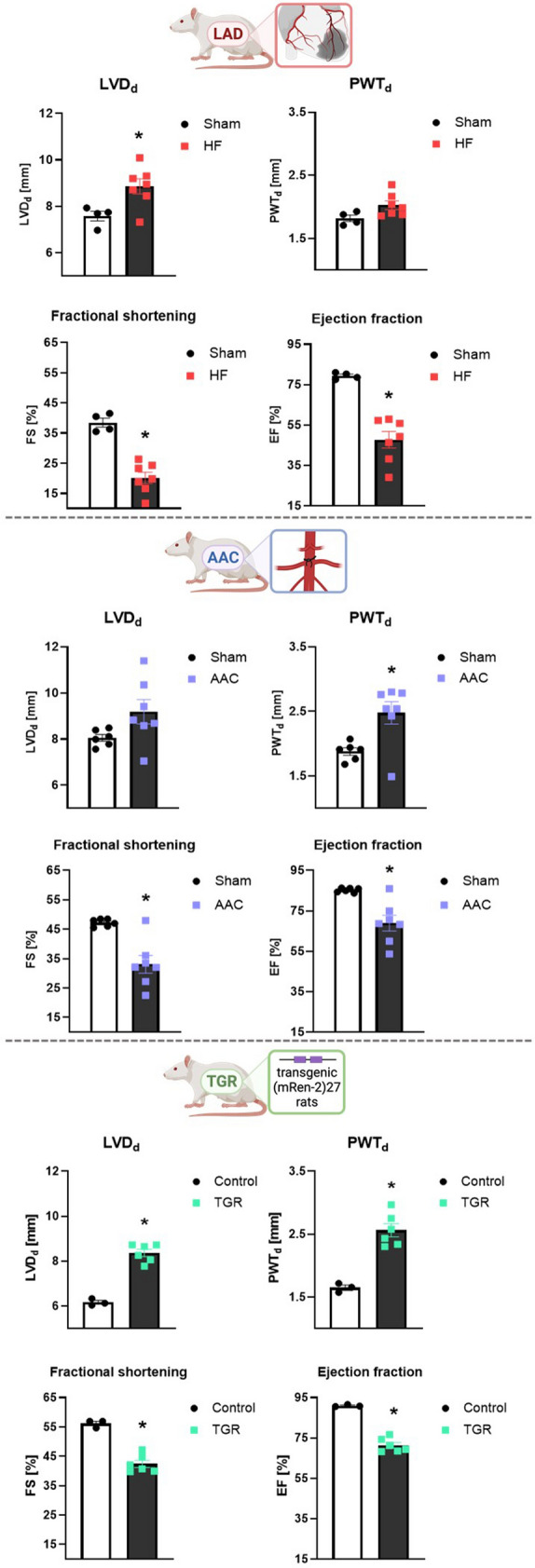
Echocardiographic assessment of cardiac function in three models of heart failure induced by myocardial infarction or pressure overload, *n* = 4–7, data are presented as mean ± SEM, unpaired t test or Mann–Whitney test, *P < 0.05

In all three animal models of HF, echocardiographic examination showed significantly reduced FS and EF advocating for the worsened LV systolic function and remodelling. In LAD and TGR animals, a significant increase in LVDd indicated LV dilatation whilst in the AAC model, there was a trend for this morphological alteration (P = 0.0805). On the other hand, both models of pressure overload-induced HF exhibited cardiac hypertrophy as evidenced by the increased PWTd. In post-MI HF, there was only a trend of increase in PWTd (P = 0.0653) (Fig. 1).

#### Molecular signalling pathways of cell death in the failing hearts of different aetiologies

##### LAD

The protein expression of the non-canonical necroptotic initiator, TRIF, was significantly upregulated in the infarcted tissue of LVs compared to both the sham group, as well as the non-infarcted LV tissue. The levels of the total and the phosphorylated form of RIP3 at Thr^231^/Ser^232^, a core component of the necroptotic signalling, were significantly upregulated in the infarcted area of the LVs whilst having only a trend of increase (~ 30%) in the non-infarcted tissue. The total and the pSer^345^-MLKL mimicked the pattern seen in the levels of (p)RIP3, thereby arguing for the promotion of necroptosis through the non-canonical pathway in conditions of 1 h ischaemia followed by 7-day recovery.

**Fig. 2:**
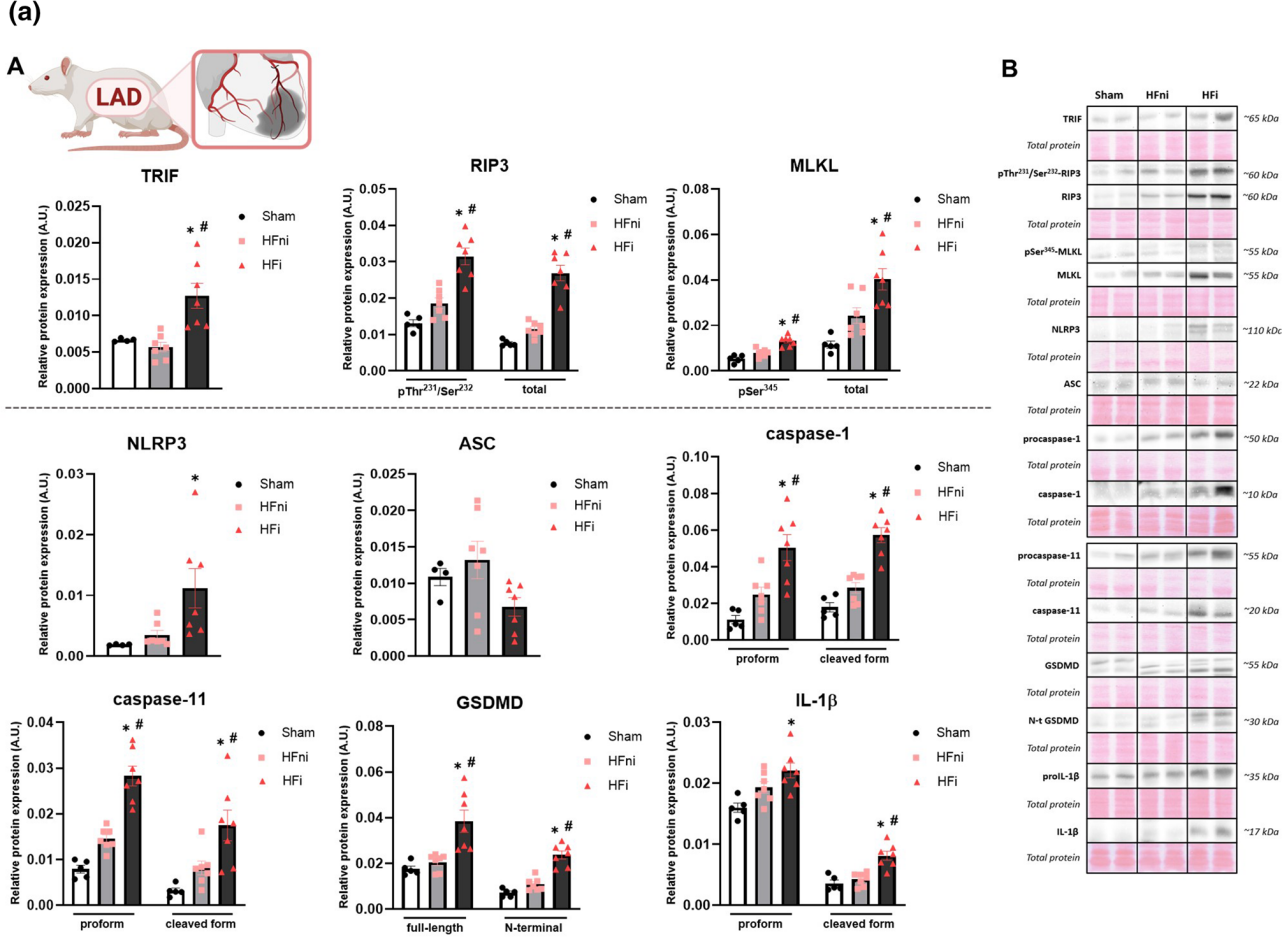

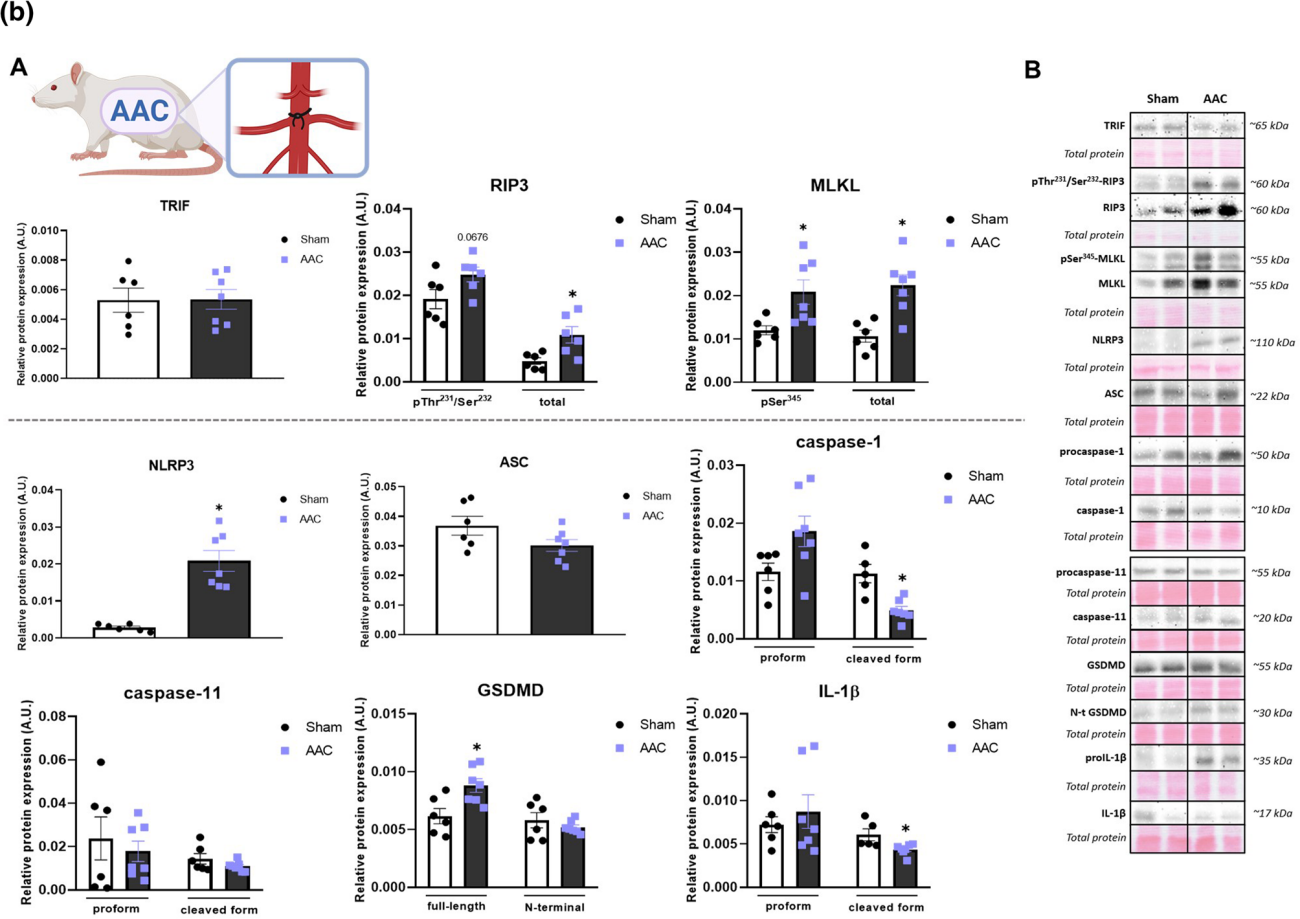

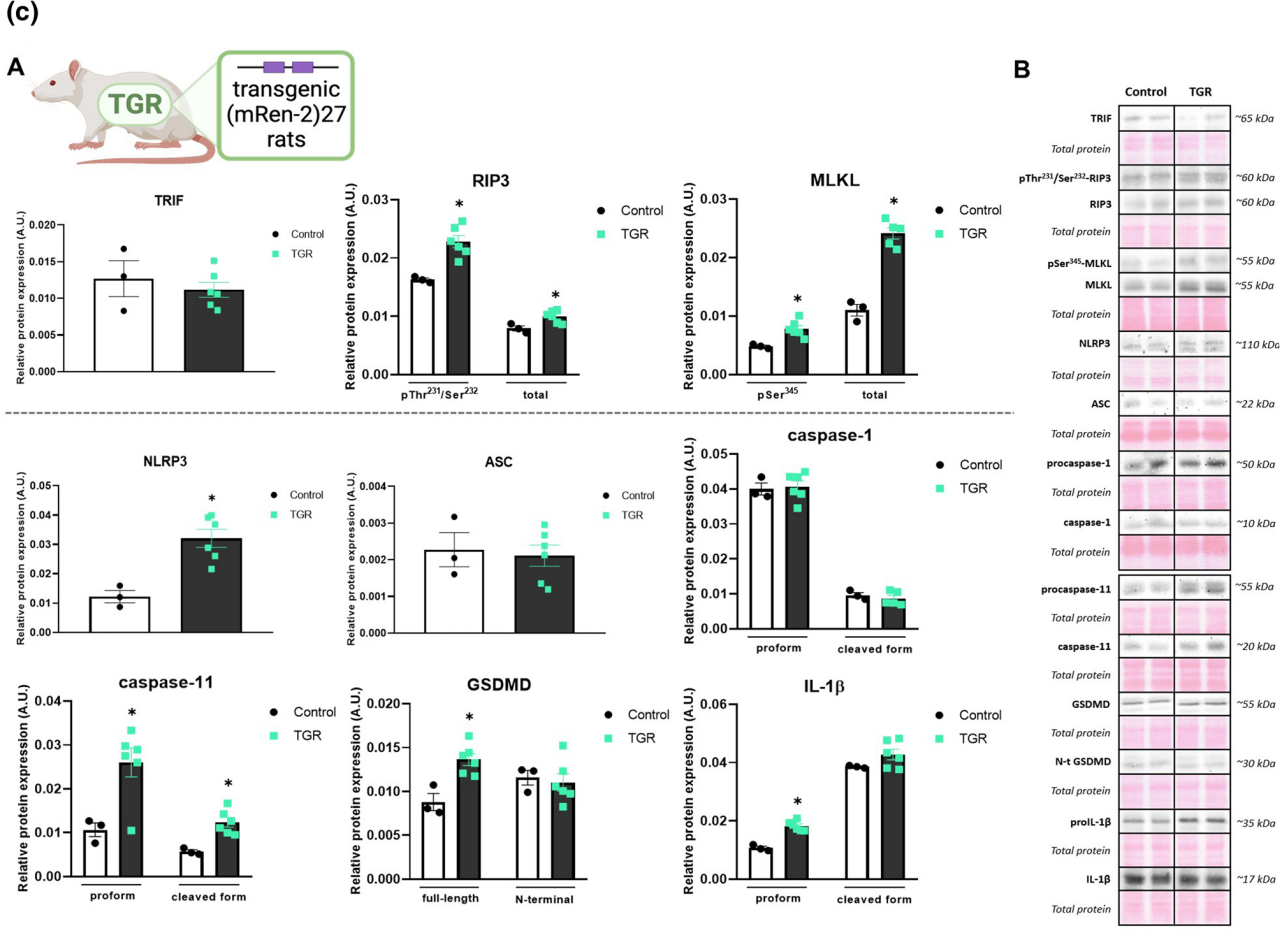
**A** Plasma membrane disrupting mechanisms due to necroptosis and pyroptosis in heart failure (HF) induced by myocardial infarction (MI). **A** Western blot analysis of MI-induced changes in the expression of necroptotic and pyroptotic proteins (TRIF, pThr^231^/Ser^232^-RIP3, RIP3, pSer^345^-MLKL, MLKL, NLRP3, ASC, (pro)caspase-1, (pro)caspase-11, (N-terminal) GSDMD, (pro)IL-1β) in Sham, non-infarcted HF (HFni) and infarcted HF (HFi) group, *n* = 4–7, data are presented as mean ± SEM, one-way ANOVA or Kruskal–Wallis test, *P < 0.05 vs Sham, #*P* < 0.05 vs HFni; **B** Representative immunoblots and total protein staining. **B** Plasma membrane disrupting mechanisms due to necroptosis and pyroptosis in heart failure (HF) induced by pressure overload due to abdominal aortic constriction (AAC). **A** Western blot analysis of AAC-induced changes in the expression of necroptotic and pyroptotic proteins (TRIF, pThr^231^/Ser^232^-RIP3, RIP3, pSer^345^-MLKL, MLKL, NLRP3, ASC, (pro)caspase-1, (pro)caspase-11, (N-terminal) GSDMD, (pro)IL-1β) in Sham and AAC group, *n* = 5–7, data are presented as mean ± SEM, unpaired t test, *P < 0.05; **B** Representative immunoblots and total protein staining. **C** Plasma membrane disrupting mechanisms due to necroptosis and pyroptosis in heart failure (HF) induced by pressure overload in transgenic (mRen-2)27 rats (TGR). **A** Western blot analysis of TGR-induced changes in the expression of necroptotic and pyroptotic proteins (TRIF, pThr^231^/Ser^232^-RIP3, RIP3, pSer^345^-MLKL, MLKL, NLRP3, ASC, (pro)caspase-1, (pro)caspase-11, (N-terminal) GSDMD, (pro)IL-1β) in Control and TGR group, *n* = 3–6, data are presented as mean ± SEM, unpaired t test, *P < 0.05; **B** Representative immunoblots and total protein staining

The protein expression of the pyroptosis initiator, NLRP3, was increased in the infarcted tissue whilst being unchanged in the non-infarcted area compared to the sham group, thus fully reflecting its mRNA levels. Interestingly, another component of the NLRP3 inflammasome—ASC, did not significantly differ between the groups. However, the pro- and cleaved forms of both caspase-1 and caspase-11 exhibited significantly elevated protein expression in the infarcted tissue of the failing hearts. The expression of both the full-length and the pore-forming N-fragment of GSDMD was increased in the infarcted tissue and unchanged in the non-infarcted zone. The protein levels of GSDMD only partially reflected its mRNA expression, which however, does not provide sufficient evidence for pyroptosis execution being proven by the N-terminal GSDMD. A similar pattern of results as detected in the other pyroptosis mediators was observed in the pro- and cleaved form of IL-1β, thereby collectively indicating the likely participation of pyroptosis in the post-MI heart damage (Fig. 2A, S1).

##### AAC

The levels of RIP3 and MLKL, both the total and phosphorylated forms, exhibited significantly higher protein expression in animals with 90-day-developing AAC-induced pressure overload compared to sham animals whilst the expression of TRIF was comparable in both groups. Even though NLRP3 and procaspase-1 protein levels were higher in AAC hearts, ASC did not show a change in protein expression. The cleaved form of caspase-1 was rather decreased due to this type of pressure overload and no changes were seen in caspase-11 expression. The level of full-length GSDMD was increased due to AAC, however, this change was not mirrored in the expression of its active, pore-forming N-fragment. In line, the active cleaved IL-1β was rather decreased in the AAC group compared to sham animals. Collectively, necroptosis, being non-dependent on TRIF, unlike pyroptosis, is likely implicated in the pathomechanisms of HF due to AAC-induced pressure overload (Fig. 2B).

##### TGR

Comparably to AAC-induced pressure overload, the LVs of 14–16-week-old TGR showed unchanged TRIF expression, whilst the level of both the total and phosphorylated form of RIP3 and MLKL were significantly elevated, thereby suggesting active necroptotic injury. In contrast, although NLRP3 expression was higher in the TGR group, pyroptosis does not appear to be responsible for the tissue damage. The main executive protein of pyroptosis, N-GSDMD, was unchanged despite the higher levels of its full-length form in TGR hearts. Likewise, neither ASC nor the pro- or cleaved caspase-1 protein expressions were changed in the TGR group. Interestingly, the levels of both the zymogen and cleaved form of caspase-11 were upregulated in TGR, however, the expression of IL-1β was unchanged (Fig. 2C).

### Effects of RIP3 inhibition on post-myocardial infarction heart failure

Because failing hearts due to MI were positive for both necroptosis and pyroptosis and because RIP3 has been suggested to function as a convergence point in their signalling pathways [23], we next investigated whether GSK'872, a RIP3 inhibitor, is cardioprotective in post-MI HF model.

#### Heart function and myocardial remodelling

**Fig. 3:**
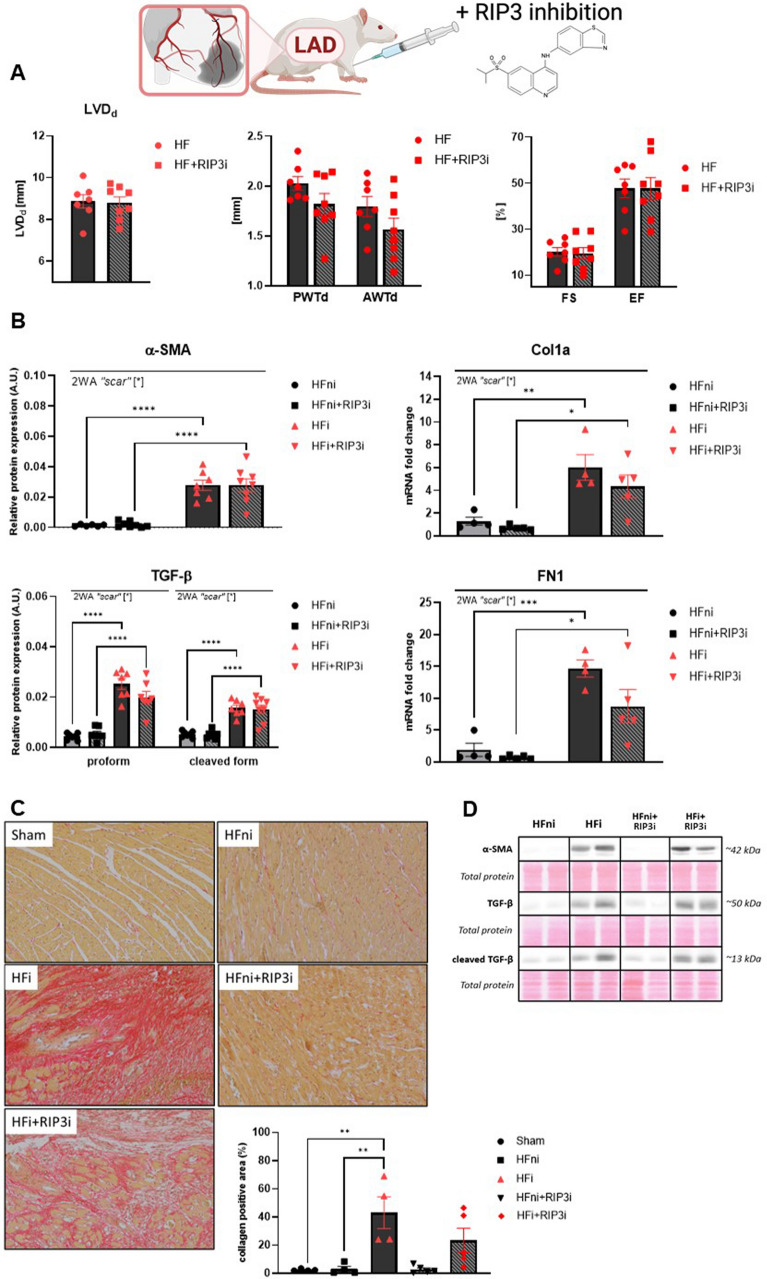
The effect of RIP3 inhibition on cardiac function and remodelling. **A** Echocardiographic assessment of cardiac function after 7 days of post-myocardial infarction recovery and the effect of RIP3 inhibition, *n* = 7–8, data are presented as mean ± SEM, unpaired t test; **B** Western blot or RT-qPCR analysis of α-SMA, TGF-β, Col1a and FN1 in non-infarcted heart failure (HFni), treated HFni (HFni + GSK´872), infarcted HF (HFi) and treated HFi (HFi + GSK´872) group, *n* = 4–8, data are presented as mean ± SEM, two-way ANOVA, *P < 0.05, **P < 0.01, ***P < 0.001, ****P < 0.0001; **C** Representative images showing collagen content and its percentage expression in Sham, non-infarcted HF (HFni), infarcted HF (HFi), treated HFni (HFni + GSK´872) and treated HFi (HFi + GSK´872) group, *n* = 4–5, data are presented as mean ± SEM, one-way ANOVA, **P < 0.01; **D** Representative immunoblots and total protein staining

RIP3 inhibition in post-MI HF did not affect any of the studied echocardiographic parameters, indicating that this treatment being administered for 7 days, was unlikely to affect cardiac function or cardiac remodelling. Similarly, the markers of fibrosis and signals for the fibroblasts to produce and remodel the extracellular matrix (alpha-smooth muscle actin (α-SMA), transforming growth factor beta (TGF-β), fibronectin-1 (FN1), collagen 1a (Col1a)) as well as collagen content were unchanged or tended to be decreased due to RIP3 inhibition (Fig. 3).

#### Necroptosis and pyroptosis signalling

**Fig. 4:**
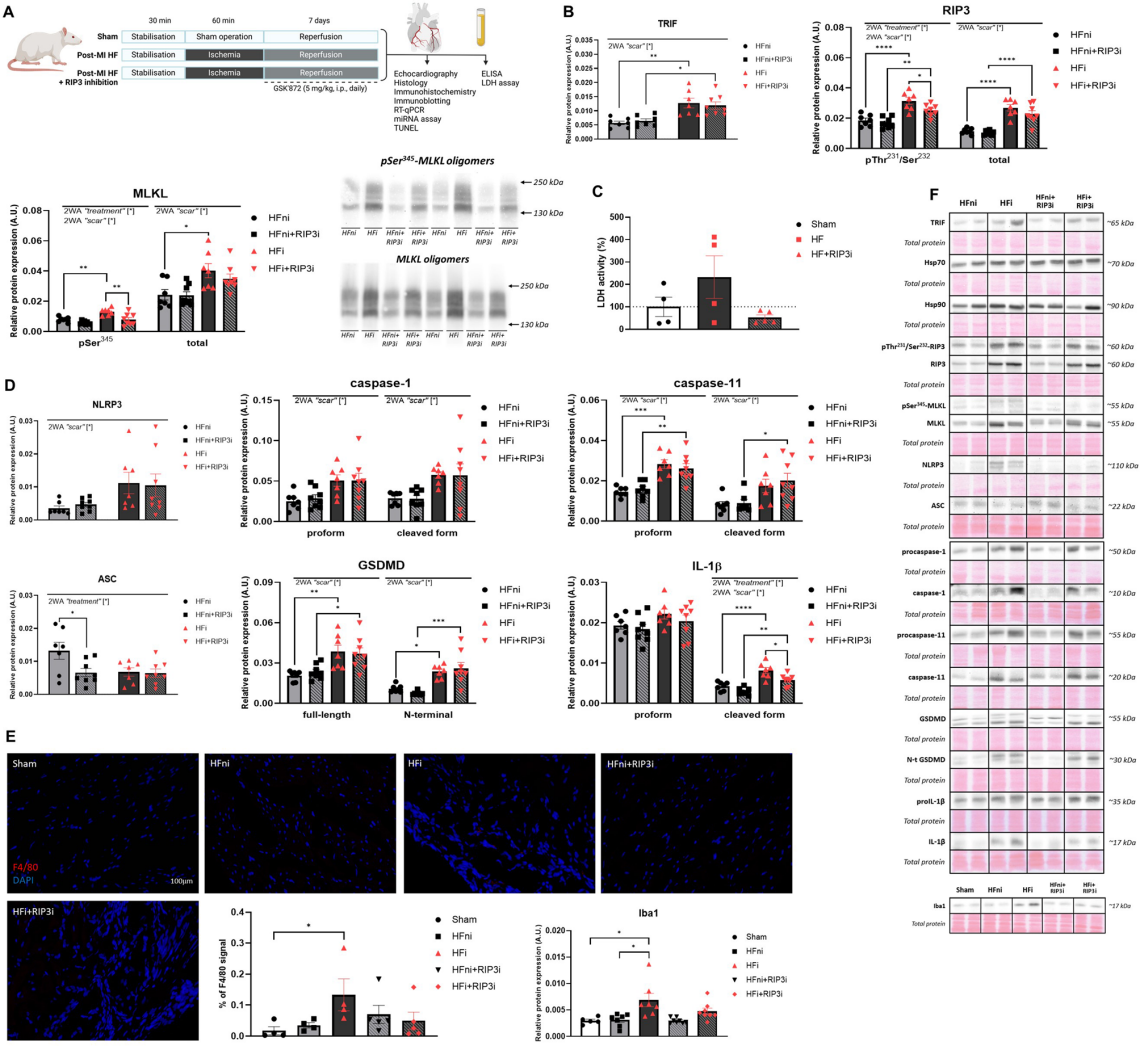
The effect of RIP3 inhibition on the plasma membrane disrupting mechanisms. **A** Schematic illustration of the experimental protocol of post-myocardial infarction (post-MI) heart failure (HF); **B** Western blot analysis of the necroptotic proteins (TRIF, pThr^231^/Ser^232^-RIP3, RIP3, pSer^345^-MLKL, MLKL, pSer^345^-MLKL and MLKL oligomeric forms) in non-infarcted HF (HFni), treated HFni (HFni + GSK´872), infarcted HF (HFi) and treated HFi (HFi + GSK´872) group, *n* = 7–8, data are presented as mean ± SEM, two-way ANOVA, *P < 0.05, **P < 0.01, ****P < 0.0001; **C** The activity of LDH after 7 days of post-MI recovery in Sham, HF and treated HF (HF + GSK´872) group, *n* = 4–5, data are presented as mean ± SEM, one-way ANOVA; **D** Western blot analysis of the pyroptotic proteins (NLRP3, ASC, (pro)caspase-1, (pro)caspase-11, (N-terminal) GSDMD, (pro)IL-1β) in non-infarcted HF (HFni), treated HFni (HFni + GSK´872), infarcted HF (HFi) and treated HFi (HFi + GSK´872) group, *n* = 7–8, data are presented as mean ± SEM, two-way ANOVA, *P < 0.05, **P < 0.01, ***P < 0.001, ****P < 0.0001; **E** Identification of macrophages infiltration—representative images showing immunofluorescence staining of F4/80 and its percentage expression and Western blot analysis of Iba1 in Sham, non-infarcted HF (HFni), infarcted HF (HFi), treated HFni (HFni + GSK´872) and treated HFi (HFi + GSK´872) group, *n* = 4–8, data are presented as mean ± SEM, Kruskal–Wallis test, *P < 0.05; (F) Representative immunoblots and total protein staining

There were no changes in the levels of necroptosis-modulating proteins, such as TRIF (Fig. 4), heat shock protein 70 (Hsp70) and Hsp90 (data not shown) due to RIP3 inhibition. The treatment significantly decreased the protein expression of both pThr^231^/Ser^232^-RIP3 and pSer^345^-MLKL in the infarcted area, but not in the non-infarcted zone. Furthermore, as can be observed from the immunoblots, RIP3 inhibition caused a noticeable decrease in the intensity of the protein signal representing the oligomers of both MLKL and pSer^345^-MLKL in the infarcted tissue. Regarding pyroptosis, the pharmacological intervention targeting RIP3 did not affect the investigated pyroptotic mediators except for decreasing the level of cleaved IL-1β in the infarcted area. In summary, RIP3 inhibition was effective in attenuating necroptosis in the infarcted area but it had no significant effect on pyroptosis signalling. This anti-necrosis-like effects of RIP3 inhibition were further confirmed by the reversal of HF-induced increased LDH levels, indicating mitigation of the plasma membrane rupture (Fig. 4). On the other hand, RIP3 inhibition unlikely exerted anti-apoptotic effects as evidenced by TUNEL assay and caspase-3 levels (Fig. S2).

To search for a cellular source of changes in these pro-death and pro-inflammatory molecular events, we analysed some markers of macrophages. RIP3 inhibition did not significantly impact the increased expression of ionised calcium-binding adapter molecule 1 (Iba1), and slightly reduced F4/80 labelling, indicating a mild mitigation of macrophage infiltration in the infarcted area (Fig. 4).

#### Alternative RIP3 targets

**Fig. 5:**
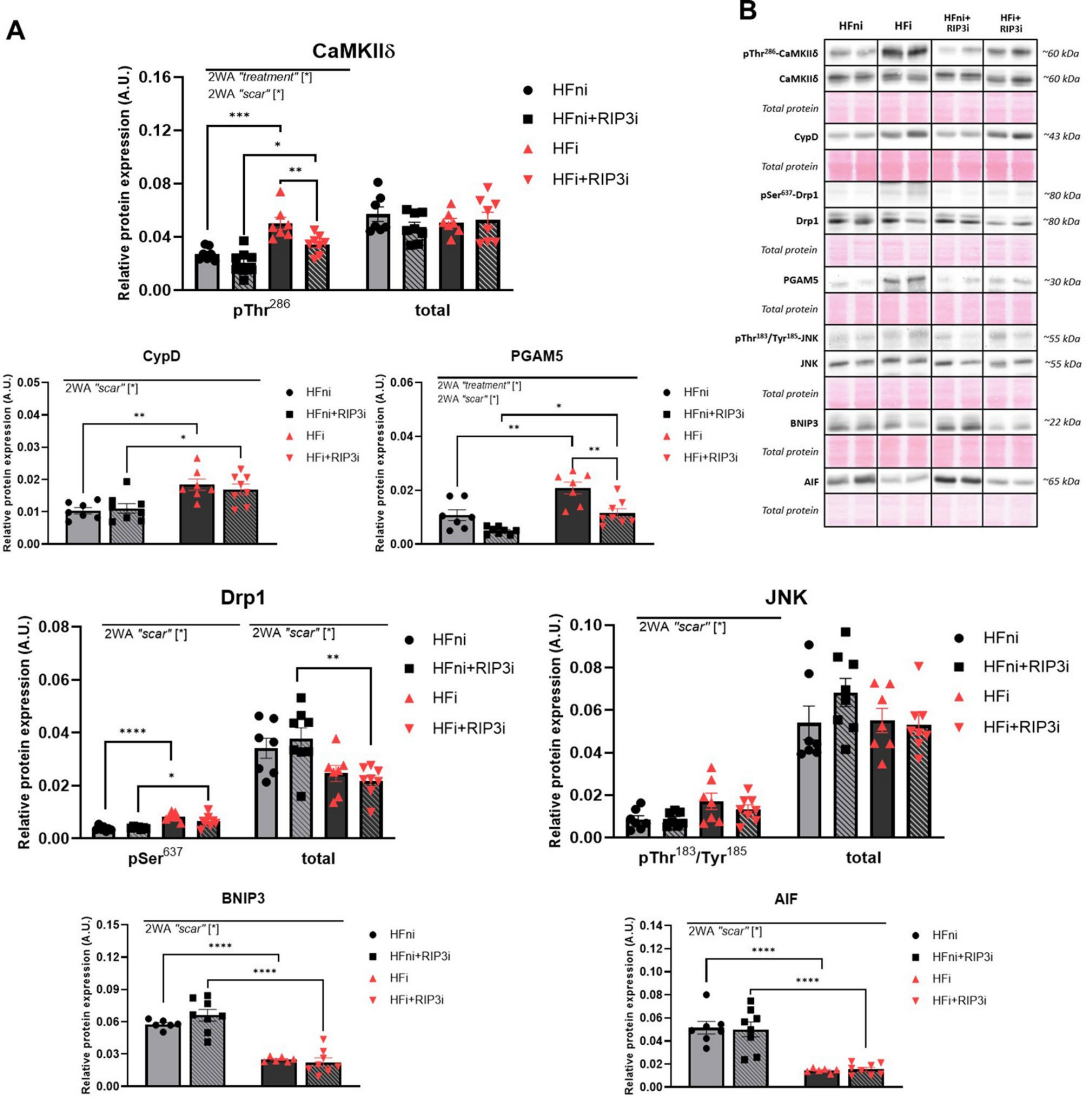
The effect of RIP3 inhibition on the alternative mechanisms of necroptosis leading to the plasma membrane rupture. **A** Western blot analysis of the proteins of alternative necroptosis signallings (pThr^286^-CaMKIIδ, CaMKIIδ, CypD, pSer^637^-Drp1, Drp1, PGAM5, pThr^183^/Tyr^185^-JNK, JNK, BNIP3, AIF) in non-infarcted heart failure (HFni), treated HFni (HFni + GSK´872), infarcted HF (HFi) and treated HFi (HFi + GSK´872) group, *n* = 6–8, data are presented as mean ± SEM, two-way ANOVA, *P < 0.05, **P < 0.01, ***P < 0.001, ****P < 0.0001; **B** Representative immunoblots and total protein staining

Because RIP3 has also been associated with other molecular targets leading to alternative, non-conventional necroptotic signalling [17], we also investigated whether such molecular axes might be relevant to our experimental conditions. Although the levels of total calcium/calmodulin-dependent protein kinase II delta (CaMKIIδ) were comparably expressed in all groups, the pThr^286^-CaMKIIδ expression was reduced due to RIP3 inhibitor in the infarcted tissue compared to the non-treated counter group. With regards to mitochondrial damage due to a pro-necroptotic environment, RIP3 inhibition did not affect the expression of a key player in mPTP opening—cyclophilin D (CypD). On the other hand, the pharmacological intervention led to a significant decrease in the levels of phosphoglycerate mutase 5 (PGAM5) in the infarcted area and a slight decrease in the non-infarcted area compared to the respective non-treated groups. However, pSer^637^-dynamin-related protein 1 (Drp1), one of the downstream targets of PGAM5, was not affected by the treatment. Furthermore, neither the RIP3─JNK─BNIP3 (c-Jun N-terminal kinase; Bcl2-interacting protein 3) nor the RIP3─AIF (apoptosis-inducing factor) signalling seemed to be affected by the RIP3 inhibitor (Fig. 5).

#### NF-κB signalling

To assess the gene regulation of RIP3-associated oxidative stress and inflammation, we focussed on nuclear factor kappa B (NF-κB) and its protein targets. RIP3 inhibition did not affect the increased levels of the total and pSer^536^-NFκB in the infarcted area. The elevated cyclooxygenase-2 (COX2) and IL-1β in the infarcted tissue was reduced by RIP3 inhibition (Fig. 4 and Fig S3). In addition, as can be observed from the immunoblot, tumour necrosis factor (TNF), another important target of NF-κB, had different banding in the infarcted zone compared to the non-infarcted tissue, advocating for the presence of the pro-inflammatory 17-kDa fragment of this cytokine in the former zone. Although the density was unable to be quantified, based on the visual analysis RIP3 inhibition unlikely significantly affected the level of this 17-kDa fragment. The treatment did not alter the levels of the cytoprotective antioxidant enzymes, such as superoxide dismutase 2 (MnSOD) and catalase (CAT), and the pro-oxidant NADPH oxidase 2 (NOX2), either (Fig. S3).

#### TLRs as the inducers of programmed necrosis and HMGB1 as a likely stimulator

The levels of toll-like receptor 2 (TLR2), TLR3 and TLR4 along with myeloid differentiation primary response 88 (MyD88), a key downstream adaptor for most TLRs, were significantly elevated in the infarcted tissue compared to the sham and non-infarcted HF group. RIP3 inhibition tended to decrease the expression of some of them, namely TLR2 and TLR3. In line with these findings, the total levels of HMGB1 serving as a potential ligand of TLRs, were elevated in the infarcted area, whilst the pharmacological treatment had no impact on the tissue levels of this DAMP.

**Fig. 6:**
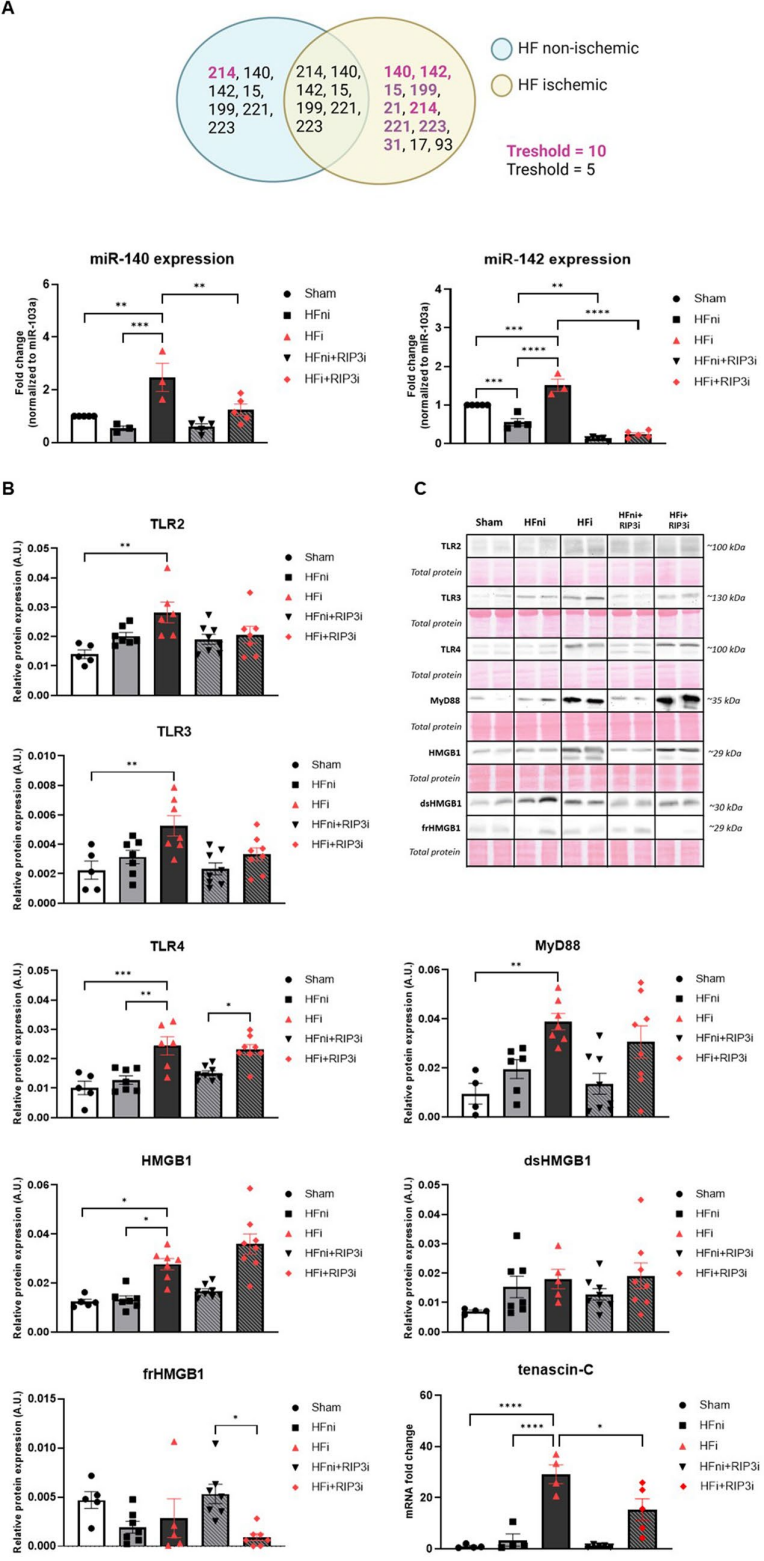
Analysis of heart failure (HF)-mediated dysregulation of miRNAs and their target proteins including upstream molecules potentially inducing necroptosis and pyroptosis and the effect of RIP3 inhibition. **A** Venn diagram showing the miRNAs being changed 5- and tenfold in non-infarcted and infarcted HF group compared to Sham group, RT-qPCR analysis of miRNA-140 and miRNA-142 in Sham, non-infarcted HF (HFni), infarcted HF (HFi), treated HFni (HFni + GSK´872) and treated HFi (HFi + GSK´872) group, *n* = 3–5, data are presented as mean ± SEM, one-way ANOVA, **P < 0.01, ***P < 0.001; ****P < 0.0001; **B** Western blot or RT-qPCR analysis of the molecules potentially associated with programmed necrosis induction (TLR2, TLR3, TLR4, MyD88, HMGB1, dsHMGB1, frHMGB1, tenascin-C) in Sham, non-infarcted HF (HFni), infarcted HF (HFi), treated HFni (HFni + GSK´872) and treated HFi (HFi + GSK´872) group, *n* = 4–8, data are presented as mean ± SEM, one-way ANOVA or Kruskal–Wallis test, *P < 0.05, **P < 0.01, ***P < 0.001, ****P < 0.0001; **C** Representative immunoblots and total protein staining

The pro-inflammatory double-stranded HMGB1 (dsHMGB1) tended to increase whilst the tissue reparation-mediating fully reduced form of HMGB1 (frHMGB1) tended to be downregulated in both damaged areas of the LVs of post-MI failing hearts. RIP3 inhibition did not reverse the expression of these HMGB1 forms in the infarcted area, however, notably it tended to upregulate the repairing frHMGB1 form in the non-infarcted zone of failing hearts. On the other hand, another ligand for TLR4, tenascin-C [55], being elevated in the infarcted zone, was significantly reduced due to RIP3 inhibition (Fig. 6).

#### Circulating levels of HMGB1

**Fig. 7:**
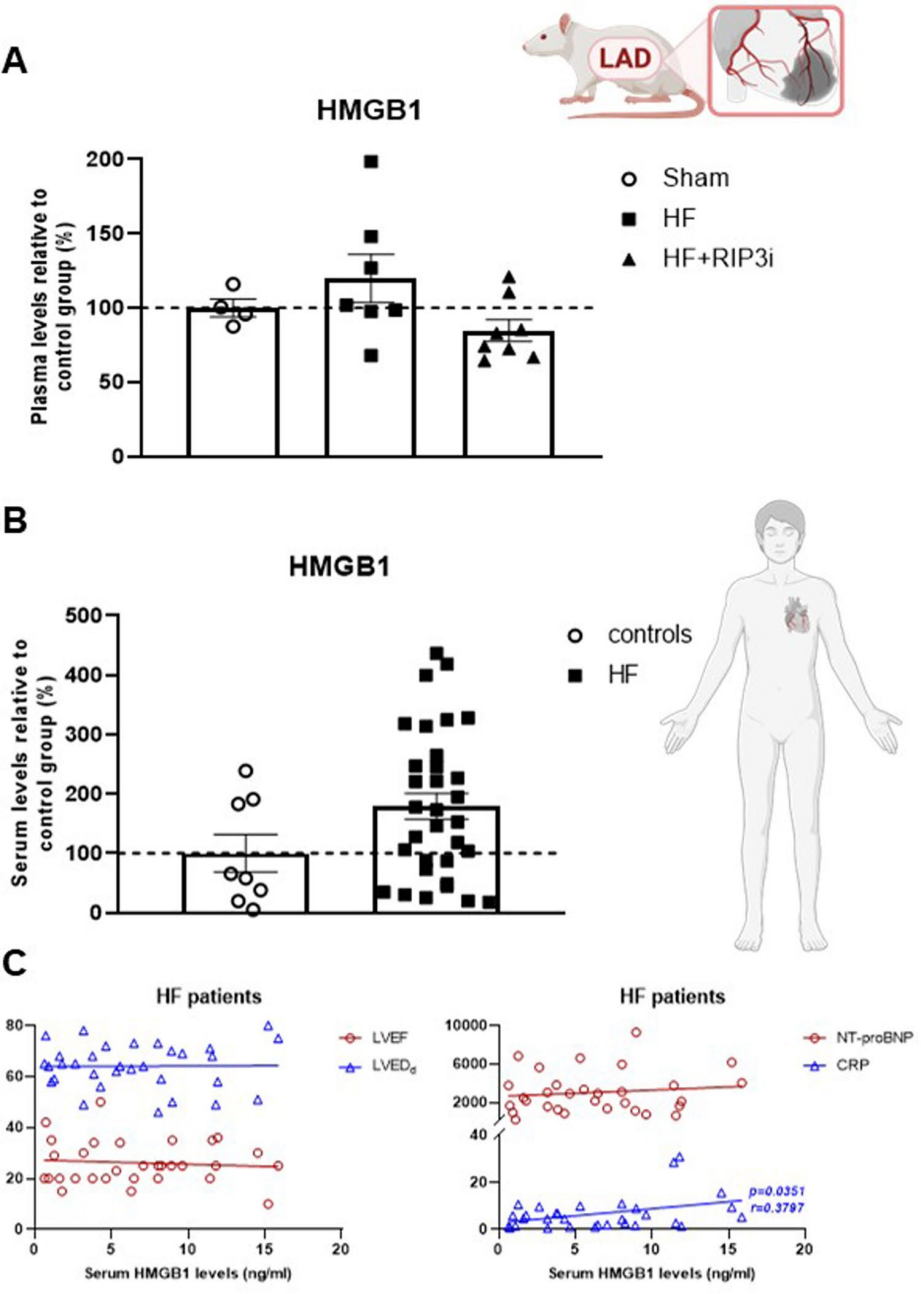
Potential consequences of the plasma membrane rupture—release of HMGB1 into the circulation. **A** The plasma levels of HMGB1 in Sham rats (*n* = 4), post-myocardial infarction heart failure (HF) rats (HF) (*n* = 7) and treated HF rats (HF + GSK´872) (*n* = 8), data are presented as mean ± SEM, one-way ANOVA; **B** The serum levels of HMGB1 in human healthy controls (*n* = 8) and patients with HF (*n* = 32), data are presented as mean ± SEM, unpaired t test; **C** Correlation between the serum HMGB1 and LVEF, LVEDd, the serum NT-proBNP and the serum CRP in HF patients (*n* = 32), Pearson correlation analysis

Given the observation of the altered myocardial levels of HMGB1 and its oligomers (dsHMGB1 and frHMGB1) in post-MI HF, we further aimed to evaluate its circulating levels. The plasma levels of HMGB1 in rats with HF were higher by about 20% compared to sham-operated animals. In contrast, RIP3 inhibition reduced the levels of this DAMP below the levels seen in healthy rats (Fig. 7).

From a translational point of view, in patients with newly diagnosed HFrEF, the serum HMGB1 levels were almost twice as high as in healthy individuals whilst also positively correlated with the serum levels of C-reactive protein (CRP), but not with the markers of cardiac function, LVEF, LVEDD and N-Terminal pro-brain natriuretic peptide (NT-proBNP) (Fig. 7). Obviously, a similar approach of an analysis of HMGB1 upon RIP3 inhibition in patients with HF could not be used from ethical concerns as the management of HF patients does not refer to RIP3 inhibition as a part of the guideline-directed therapy.

### miRNA tissue levels

The miRCURY LNA miRNA PCR assay analysis revealed a wide profile of miRNAs being changed in the LVs of rats with post-MI HF compared to the sham group. A 5- and tenfold change in the levels of miRNA-140, -142, -15, -199, -221 and -223 was found in the non-infarcted and infarcted areas of the failing hearts, respectively. Furthermore, both tissue zones exhibited a tenfold increase in miRNA-214 levels (Fig. 6). Using an online available platform (https://www.targetscan.org/) biological targets of these miRNAs were predicted and the particular miRNAs being associated with the proteins of our interest were further rechecked by RT-qPCR analysis. HF-induced dysregulation of miRNA-140 and -142 was confirmed, and RIP3 inhibition was found to normalise their levels (Fig. 6).

## Discussion

In the present study, necrosis-like death modes, and pro-inflammatory molecular events have been examined to reveal the potential underlying pathomechanisms of HF of various origins. Namely, post-MI HF and HF due to pressure overload, induced by AAC or genetically by the overexpression of the Ren-2 gene, were studied. Several novelties have been identified: i) both non-canonical necroptosis and pyroptosis signalling likely contribute to the myocardial tissue damage and remodelling due to MI whilst only the former cell death mode is activated in the models of HF due to pressure overload, ii) the pro-inflammatory receptors of the TLR family might play an important role in programmed necrosis in post-MI HF, iii) HMGB1 and its mono-/oligomers are increased in the post-MI failing hearts, iv) the increased tissue amount of HMGB1 is likely reflected in the circulating HMGB1 levels since they were found to be elevated also in the HF patients, thereby highlighting the clinical relevance of this pro-necro(pto)tic DAMP. Furthermore, we found for the first time that short-term RIP3 inhibition by GSK'872 in post-MI HF is able to mitigate necroptotic pathway, including reduction of the formation of the cytotoxic MLKL oligomers, and to mitigate the local tissue inflammation possibly due to the prevention of macrophage infiltration. This anti-necroptotic approach also normalised the higher levels of miRNA-140 and -142 being associated with pro-death, pro-inflammatory events in post-MI HF. Lastly, RIP3 inhibition tended to reduce collagen and fibrosis markers and signals for fibroblasts, with no apparent impact on LV systolic function (Summary Figure).

The LVs of failing hearts due to both MI and pressure overload were positive for the main necroptosis markers, pRIP3 and pMLKL, thereby indicating the pro-necroptotic cell-damaging environment under such conditions of heart damage. These findings are fully in accordance with findings on human failing hearts being of comparable aetiology, such as ischaemic and dilated cardiomyopathy [39]. Likewise, the evidence of necroptosis in 7-day-post-MI HF model is partially in line with our previous study employing a chronic, 42-day-post-MI HF model [24]. Indeed, both the infarcted and non-infarcted areas exhibited active necroptosis evidenced by the higher levels of pMLKL and the presence of MLKL oligomers, whilst in the latter zone of the more advanced HF, there were higher levels of RIP3 and pro-inflammatory environment, only. These seemingly contradictory data might indicate that during the first 7 days of post-MI recovery, necroptotic cell loss is predominant and thus likely affects the whole LV. On the other hand, in the following phase of progressive myocardial damage, RIP3 keeps the MLKL activation in the infarcted area only and rather mediates MLKL-independent pro-inflammatory damage in the non-infarcted area. It is worth noting that all above-mentioned studies on HF have focussed solely on the canonical RIP1-dependent pathway of necroptosis. Herein, we also focussed on the likely involvement of the non-canonical pathway of necroptosis. We showed for the first time that TRIF is upregulated in post-MI HF, indicating its upstream position to the activated RIP3─MLKL axis. In contrast, TRIF levels were unchanged in adult rats with HF due to neonatal AAC-induced pressure overload and genetically induced alterations in the Ren-2 gene. Thus, it is likely that the pro-necroptotic RIP3─MLKL axis in these pressure-induced HF models was activated differently. Contrary to our findings, Bettink et al. indicated an important role of TRIF in transverse aortic constriction (TAC)-induced myocardial remodelling, which was, however, associated with inflammation and apoptosis rather than necroptosis induction [6]. Such discrepancies might be attributable to the differences in the experimental model used. In a study by Bettink et al., aortic constriction was induced in adult rats, whilst here we employed a model of AAC-induced pressure overload in 2-day-old rats resulting in progressive phenotype development over 90 days, which more likely resembles the development of pressure overload-induced heart damage in the human population [22].

Since there is evidence for a link between necroptosis and pyroptosis with a crucial role of RIP3 [23], we next investigated the signalling of pyroptosis as another potential RIP3-dependent pathway promoting myocardial damage due to HF. Several experimental and clinical reports showed that pyroptosis contributes to myocardial inflammation and adverse remodelling of the heart, whilst its pharmacological inhibition is cardioprotective [3, 13, 54]. In our settings of post-MI HF, only the infarcted areas of the LVs were positive for pyroptosis execution evidenced by NLRP3-mediated cleavage of caspase-1 and IL-1β, and most importantly the cytotoxic N-GSDMD. This pro-inflammatory environment in the infarcted area 1 week after LAD release is likely caused by the macrophage infiltration. Contrary to such findings, in our previous chronic post-MI HF model, cleavage of caspase-1 and IL-1β was present in both LV areas, thereby indicating the expansion of the inflammation in the surrounding tissue [24]. In other words, in the first week after MI, despite necroptosis, pyroptosis is not executed in the non-infarcted LV tissue but becomes relevant only in the later stages of post-MI HF, promoting further progressive myocardial damage. It is, however, noteworthy, that in our previous study, we did not examine the levels of the most relevant pyroptotic marker, N-GSDMD, and thus it is not clear whether the activation of the caspase-1–IL-1β axis proceeded to pyroptotic loss of cardiac cells or pro-inflammatory tissue damage only. Of note, besides the canonical pyroptosis markers, we also found the levels of caspase-11, known to promote non-canonical signalling, to be increased due to post-MI HF. This is consistent with the studies showing that caspase-11-mediated non-canonical pathway also contributes to myocardial I/R injury [36]. On the other hand, even though there was an upregulation of NLRP3 expression in both models of pressure-overload-induced HF used in this study, pyroptotic cell loss was absent based on the unchanged levels of cleaved GSDMD. However, it should be mentioned that some studies implicating a TAC-induced HF model have reported divergent findings on pyroptosis, which can be related to experimental conditions [44, 50]. Collectively, it is very likely that different pro-inflammatory, pro-cell death mechanisms are activated in various aetiologies of HF. Although this partial conclusion is based mainly on Western blott results, this methodological approach, providing the evidence on the activation of pro-death signalling and on building of oligomeric structures, fully meets the requirements of the current guideline for the evaluation of myocardial cell death forms. It is further supported by LDH assay referring to the disruption of the plasma membrane and thus non-specific evaluation of necrosis-like cell death modes [28].

As a follow-up investigation, we performed a pharmacological study exploring the capability of RIP3 inhibition to affect the pro-death, RIP3-associated molecular events in HF due to MI. By this approach, we also tested a hypothesis that RIP3 promotes pyroptotic cell death besides necroptotic one. Even though the administration of GSK'872 over 7 days of post-MI recovery had no significant impact on the cardiac function and structure assessed by echocardiographic measurements, this pharmacological modulator had multiple effects on the studied molecular pathways. First, as could be expected, it decreased the protein levels of the core necroptotic mediators, pRIP3 and pMLKL. In addition, the RIP3 inhibitor also limited the oligomerization of MLKL, thereby arguing for the retardation of necroptotic cell loss in the treated post-MI HF. Although there are certain protocol differences, our findings are in line with the results of a study using the same RIP3 inhibitor given 48 h after the ischaemic insult of the brain [47]. Likewise, the retardation of necroptotic signalling was observed in the liver treated by GSK'872 before ischaemia induction [46]. The findings presented herein are original because, according to our best knowledge, the RIP3 inhibitor was not previously used in such a clinically relevant model of heart damage. Likewise, this is the first report showing its ability to affect the non-canonical pathway of necroptosis, which might be even more important than the canonical one. Furthermore, since RIP3 has been implicated in several alternative, non-conventional necroptotic pathways [17], we evaluated whether such molecular axes are affected by GSK'872. It decreased the levels of pCaMKII and PGAM5 in the infarcted area, indicating the likely limitation of mitochondria-related pro-necroptotic signalling. Consistently, Zhang et al. also found that the administration of GSK'872 was able to mitigate the phosphorylation of CaMKII in an in vivo model of TAC-induced myocardial fibrosis [52]. However, in our study, no effect was observed on the expression of other mitochondria-associated pro-necroptotic markers such as (p)Drp1, (p)JNK, BNIP3 or CypD. These findings might suggest that RIP3 inhibition mitigates ROS production by decreasing pCaMKII and PGAM5 but had no overall effect on the mitochondria. Interestingly, in a global cerebral I/R damage model, the application of GSK'872 reduced cerebral inflammation by reducing the expression of JNK [18]. Although in our hands, both the total and pJNK levels were unaffected by RIP3 inhibition, such treatment limited the expression of COX2 in the infarcted area, indicating its potential anti-inflammatory effect due to the impact on different molecular pathways. In line with these effects and the effects on IL-1β, the macrophage labelling and infiltration assessed by F4/80 and Iba1 also tended to be reduced by RIP3 inhibitor, thereby collectively advocating for its anti-inflammatory action. On the other hand, even though RIP3 can also be involved in the pyroptotic signalling [23], the inhibition of this kinase in this study did not mitigate pyroptotic death. Thus, it can be proposed that other more specific anti-pyroptotic approaches might be needed to potentiate the RIP3-inhibition mediated cardioprotection in post-MI HF. Second, GSK'872 mitigated the HF-induced dysregulation in the levels of miRNA-140 and miRNA-142, which are considered to be HMGB1-associated molecules.

To gain a deeper insight into these findings and examine underlying disturbances in the inflammatory state of post-MI HF, we examined the levels of TLRs as well as the levels of a DAMP candidate—HMGB1 which might serve as a ligand to these receptors. We evaluated for the first time the levels of oligomeric structures of HMGB1, namely the pro-inflammatory, immune cells attracting dsHMGB1 and the frHMGB1 which has been suggested to be responsible rather for tissue reparation [12, 34]. RIP3 inhibition did not affect the levels of dsHMGB1. Interestingly, frHMGB1 seemed to be increased by RIP3 inhibitor in the non-infarcted zone and decreased in the infarcted LV tissue, indicating its action also on the immune cells-mediated reparative processes. However, it should be mentioned that the origin and the redox state of circulating as well as tissue HMGB1 is currently only partially described, and there is only a very limited number of studies on this topic. Ferrara et al. have found that infiltrating leukocytes produce altered levels of dsHMGB1 and frHMGB1, thus mediating acute damage to the skeletal muscle, spleen, and liver [12]. With regards to the receptors associated with HMGB1, we found that the infarcted tissue exhibited the increased expression of TLR2, TLR3, TLR4 and MyD88, which is in line with the growing evidence about the role of inflammation under HF conditions [40, 48, 49]. Although RIP3 inhibitor non-significantly reduced TLR2 and TLR3 expression, a ligand of TLR4—tenascin-C was downregulated by this pharmacological approach.

The potential clinical implication of this study was highlighted by assessing the circulating levels of HMGB1 as a novel HF biomarker. In the rats with failing hearts due to MI, the plasma HMGB1 levels were increased by approximately 20%. At the same time, the RIP3 inhibitor reversed these levels, supporting our findings that GSK'872 is able to reduce the necrosis-like cell death and subsequent release of DAMPs. The two-fold increase in the circulating levels of HMGB1 was also found in patients with diagnosed HFrEF of various aetiology and severity. These findings are in accordance with the previous studies which reported that the higher circulating levels of HMGB1 correlate positively with left ventricular end-diastolic and end-systolic volumes, NT-proBNP levels, NYHA class, and white blood cell count, whilst inversely correlating with LVEF [27, 42, 45]. In our hands, the increased HMGB1 correlated with the serum CRP levels. Given this fact, it can be noted that in these patients with HF, the serum RIP3 levels also tended to be increased [19] thereby supporting the relevance of RIP3-mediated necroptosis damage and inflammation under the conditions of HF.

**Fig. 8:**
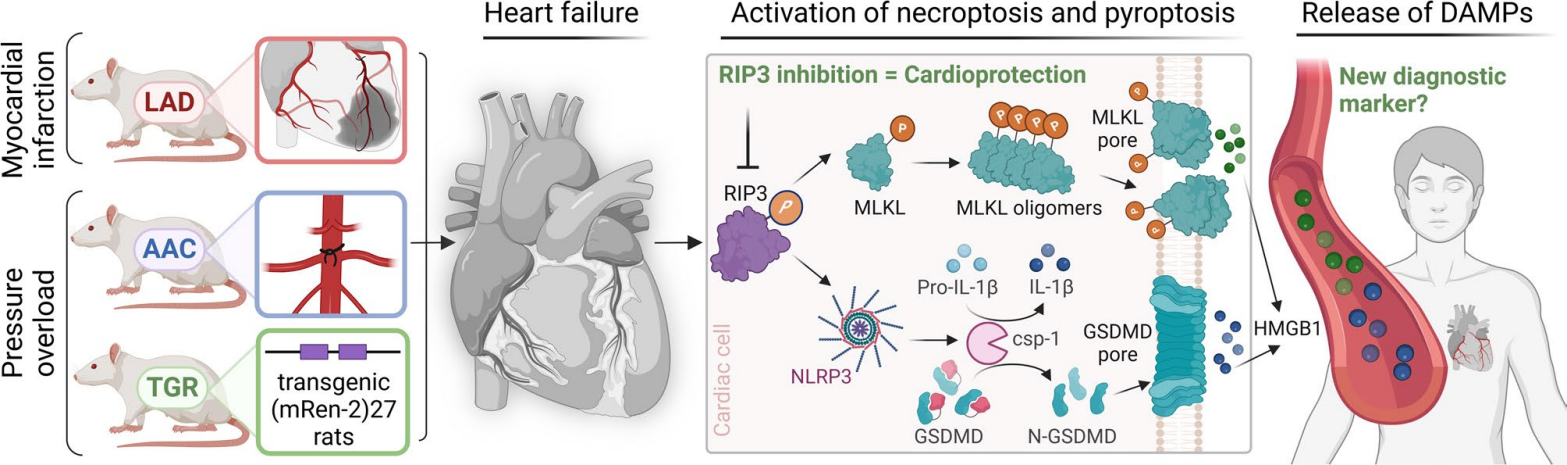
The regulated necrosis-like cell death forms in heart failure due to myocardial infarction or pressure overload, the potential consequences of the plasma membrane rupture and the effect of RIP3 inhibition

In conclusion, we showed that the cardiac damage due to post-MI HF is amplified by the interplay of both non-canonical necroptosis and pyroptosis, Whilst only the former necrosis-like cell death is likely responsible for the loss of cardiac cells in pressure overload-mediated HF. HMGB1 might play an important role in promoting pro-cell death and pro-inflammatory events in post-MI HF and might serve as a supportive marker of HFrEF. RIP3 inhibition in post-MI HF prevented the execution of necroptotic injury and local tissue inflammation likely due to retardation of macrophage infiltration and lower release of DAMPs. It tended to affect collagen content and pro-fibrotic molecular mechanisms without apparent effects on cardiac structure and function. Chronic treatment and/or multiple pharmacological approaches targeting other mechanisms alongside RIP3 itself might be needed to achieve stronger cardioprotection (Fig. 8).

## Supplementary Information

Below is the link to the electronic supplementary material.

Supplementary file1 (DOCX 2938 KB)

Supplementary file1 (PNG 2012 KB)

## Data Availability

All the data of this study are available from the corresponding author upon reasonable request.
