# Supplementary material for: Necrosis-like cell death modes in heart failure: the influence of aetiology and the effects of RIP3 inhibition
Source: Basic Res Cardiol. 2025 Mar 15;120(2):373–92. doi: 10.1007/s00395-025-01101-4 (PMC11976840; doi:10.1007/s00395-025-01101-4)
Supplement: Supplementary file 1 — Supplementary file1 (DOCX 2938 KB) [file 395_2025_1101_MOESM1_ESM.docx]

**SUPPLEMENTARY MATERIAL**

**Necrosis-like cell death modes in heart failure: the influence of aetiology and the effects of RIP3 inhibition**

Izabela Jarabicová^1^, Csaba Horváth^1^, Jaroslav Hrdlička^2^, Almos Boroš^2^, Veronika Olejníčková^2,3^, Eva Zábrodská^3^, Soňa Štemberková Hubáčková^4,5^, Hana Mauer Šutovská^6^, Ľuboš Molčan^6^, Libor Kopkan^4^, Martin Chudý^7^, Branislav Kura^8^, Barbora Kaločayová^8^, Eva Goncalvesová^7^, Jan Neckář^2^, Michal Zeman^6^, František Kolář^2^, Adriana Adameová^1,8^

1 Faculty of Pharmacy, Department of Pharmacology and Toxicology, Comenius University, Bratislava, Slovak Republic

2 Institute of Physiology of the Czech Academy of Sciences, Prague, Czech Republic

3 Institute of Anatomy, First Faculty of Medicine, Charles University, Prague, Czech Republic

4 Institute for Clinical and Experimental Medicine, Prague, Czech Republic

5 Institute of Biotechnology, Czech Academy of Sciences, Prague, Czech Republic

6 Faculty of Natural Sciences, Department of Animal Physiology and Ethology, Comenius University, Bratislava, Slovak Republic

7 Department of Cardiology, Faculty of Medicine, Comenius University and National Cardiovascular Institute, Bratislava, Slovak Republic

8 Centre of Experimental Medicine, Institute for Heart Research, Slovak Academy of Sciences, Bratislava, Slovak Republic

Correspondence: prof. PharmDr. Adriana Duriš Adameová, PhD., FIACS; Faculty of Pharmacy, Department of Pharmacology and Toxicology, Comenius University, Odbojárov 10, 832 32 Bratislava, Slovak Republic; email: adameova@fpharm.uniba.sk; Tel. +421 2 50117 366; Fax +421 2 50 117 100

**Supplementary figures**

**Fig. S1** mRNA levels of NLRP3 and GSDMD and the effect of RIP3 inhibition. (A) RT-qPCR analysis of NLRP3 in non-infarcted heart failure (HFni), infarcted HF (HFi), treated HFni (HFni+GSK´872) and treated HFi (HFi+GSK´872) group, n=4-6, data are presented as mean±SEM, two-way ANOVA; (B) RT-qPCR analysis of GSDMD in non-infarcted HF (HFni), infarcted HF (HFi), treated HFni (HFni+GSK´872) and treated HFi (HFi+GSK´872) group, n=4-6, data are presented as mean±SEM, two-way ANOVA, *P<0.05


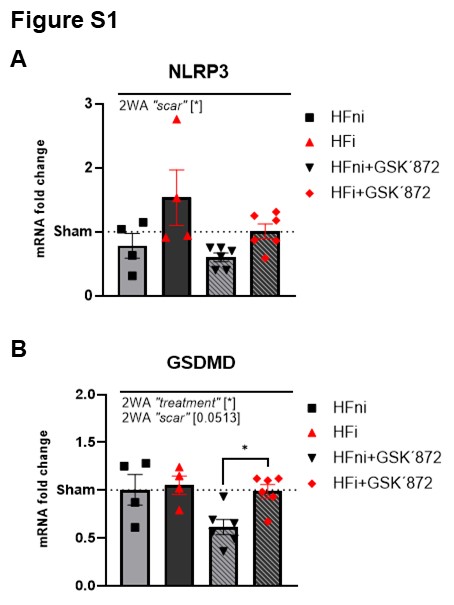


**
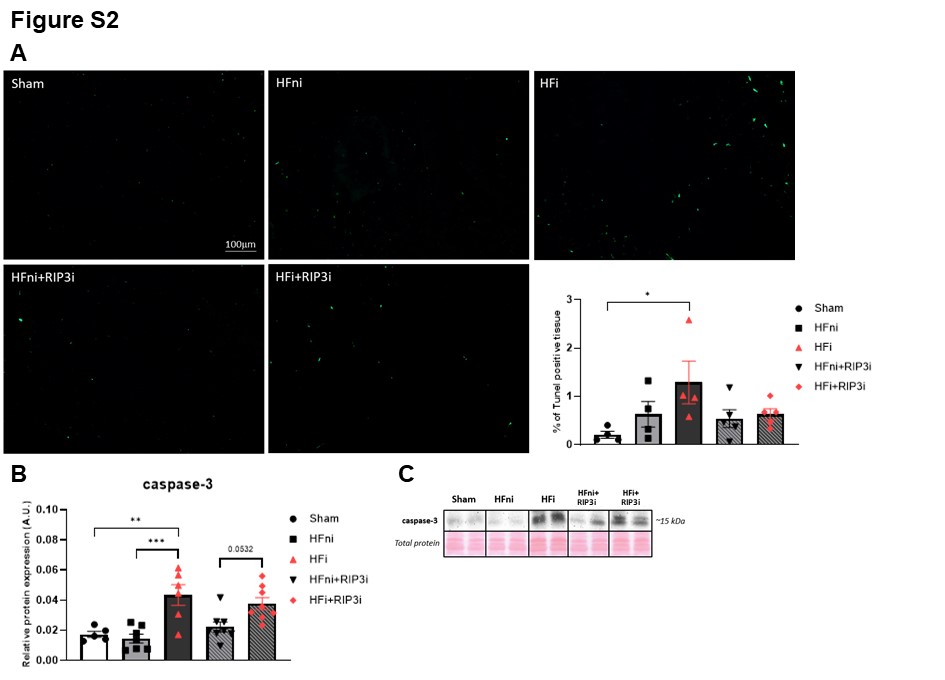
Fig. S2** Assessment of apoptosis in post-myocardial infarction heart failure (HF) and the effects of RIP3 inhibition. (A) TUNEL staining and percentage expression of TUNEL-positive cells in Sham, non-infarcted HF (HFni), infarcted HF (HFi), treated HFni (HFni+GSK´872) and treated HFi (HFi+GSK´872) group, n=4-5, data are presented as mean±SEM, one-way ANOVA, *P<0.05; (B) Western blot analysis of proapoptotic caspase-3 in Sham, non-infarcted HF (HFni), infarcted HF (HFi), treated HFni (HFni+GSK´872) and treated HFi (HFi+GSK´872) group, n=5-8, data are presented as mean±SEM, one-way ANOVA, **P<0.01, ***P<0.001; (C) Representative immunoblot and total protein staining

**
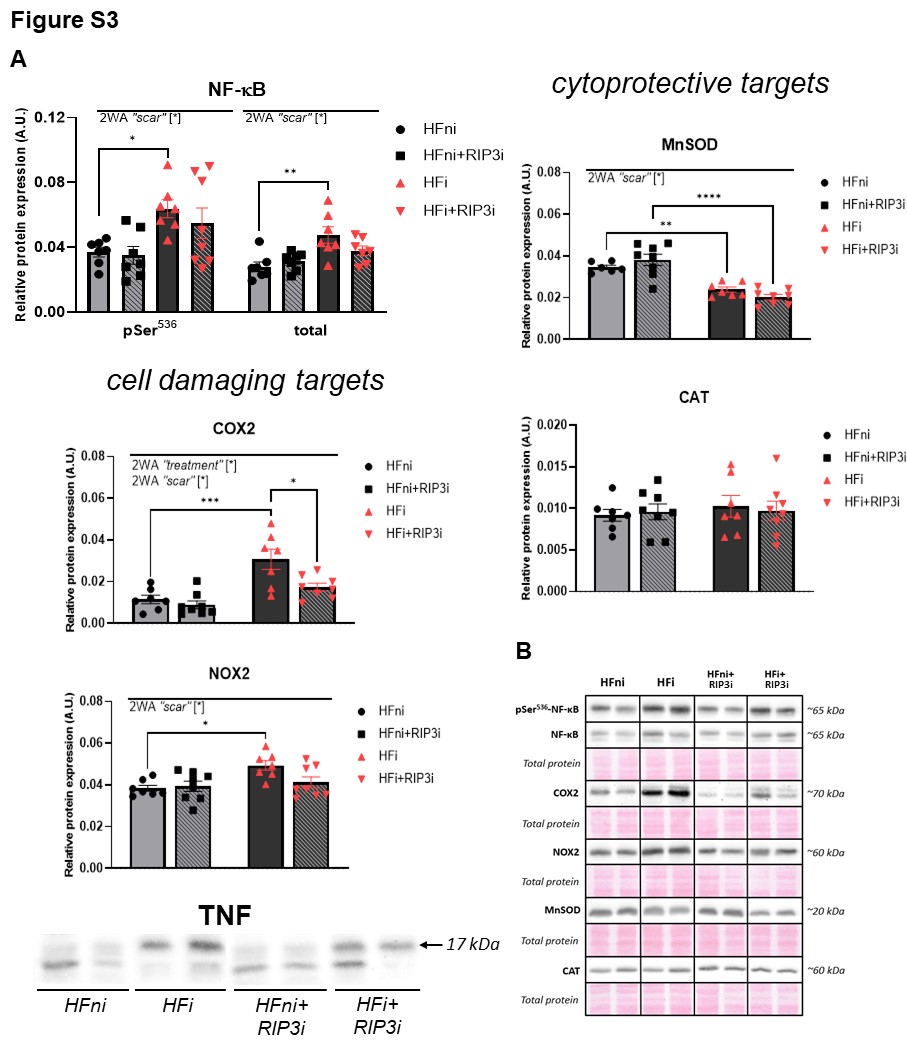
Fig. S3** Effect of RIP3 inhibition on the expression of NF-κB and its cell damaging and cytoprotective targets. (A) Western blot analysis of pSer^536^-NF-κB, NF-κB, COX2, NOX2, TNF, MnSOD and CAT in non-infarcted heart failure (HFni), treated HFni (HFni+GSK´872), infarcted HF (HFi) and treated HFi (HFi+GSK´872) group, n=6-8, data are presented as mean±SEM, two-way ANOVA, *P<0.05, **P<0.01, ***P<0.001, ****P<0.0001; (B) Representative immunoblots and total protein staining

**Supplementary tables**

**Table S1** Primary antibodies used for the detection of proteins presented in the main text of the manuscript

| **1° antibody** | **Catalogue number** | **Manufacturer** |
| --- | --- | --- |
| α-SMA | M0851 | Dako, USA |
| AIF | #4642 | Cell Signaling Technology, USA |
| ASC | #67824 | Cell Signaling Technology, USA |
| BNIP3 | #3769 | Cell Signaling Technology, USA |
| CaMKII | ab181052 | Abcam, UK |
| caspase-1 | ab179515 | Abcam, UK |
| caspase-3 | #9662 | Cell Signaling Technology, USA |
| caspase-11 | #14340 | Cell Signaling Technology, USA |
| CAT | #14097 | Cell Signaling Technology, USA |
| COX2 | #12282, | Cell Signaling Technology, USA |
| CypD | #78247 | Cell Signaling Technology, USA |
| Drp1 | #5391 | Cell Signaling Technology, USA |
| GSDMD | #46451 | Cell Signaling Technology, USA |
| HMGB1 | ab18256 | Abcam, UK |
| Hsp70 | #4872 | Cell Signaling Technology, USA |
| Hsp90 | #4877 | Cell Signaling Technology, USA |
| Iba1 | ab108539 | Abcam, UK |
| IL-1β | ab9722 | Abcam, UK |
| JNK | #9252 | Cell Signaling Technology, USA |
| MLKL | ab243142 | Abcam, UK |
| MyD88 | #4283 | Cell Signaling Technology, USA |
| NF-κB | #8242 | Cell Signaling Technology, USA |
| NLRP3 | ab263899 | Abcam, UK |
| NOX2 | ab129068 | Abcam, UK |
| PGAM5 | ab244218 | Abcam, UK |
| pSer637-Drp1 | #4867 | Cell Signaling Technology, USA |
| pSer345-MLKL | MABC-1158 | Merck, Germany |
| pSer536-NF-κB | #3033 | Cell Signaling Technology, USA |
| pThr286-CaMKII | ab171095 | Abcam, UK |
| pThr138/Tyr185-JNK | #9255 | Cell Signaling Technology, USA |
| pThr231/Ser232-RIP3 | #91702 | Cell Signaling Technology, USA |
| RIP3 | #15828 | Cell Signaling Technology, USA |
| SOD2 (MnSOD) | #13141 | Cell Signaling Technology, USA |
| TGF-β | ab179695 | Abcam, UK |
| TLR2 | ab209217 | Abcam, UK |
| TLR3 | ab13915 | Abcam, UK |
| TLR4 | ab217274 | Abcam, UK |
| TNF | ab66579 | Abcam, UK |
| TRIF | #PA5-23467 | Invitrogen, Waltham, USA |

**Table S2** Thermal programme for miRCURY LNA SYBR Green PCR used for miRCURY LNA miRNA PCR Assay panel

| **Step** | **Temperature** | **Time** | **Cycles** |
| --- | --- | --- | --- |
| PCR initial heat activation | 95 °C | 2 min. |  |
| Denaturation | 95 °C | 10 s. | 40x |
| Annelation | 56 °C | 60 s. |  |

**Table S3** Thermal programme for RT-qPCR miRNA analysis used for analysing of miRNA-140-5p and miRNA-142-3p

| **Step** | **Temperature** | **Time** | **Cycles** |
| --- | --- | --- | --- |
| Polymerase activation | 95 °C | 20 s. |  |
| Denaturation | 95 °C | 3 s. | 40x |
| Annelation | 60 °C | 30 s. |  |

**Table S4** List of measured miRNAs and their mature sequences

| **miRNA name** | **Species** | **Mature sequence** |
| --- | --- | --- |
| miRNA-140-5p | *Rattus norvegicus* | CAGUGGUUUUACCCUAUGGUAG |
| miRNA-142-3p | *Rattus norvegicus* | UGUAGUGUUUCCUACUUUAUGGA |
| miRNA-103a-3p | *Rattus norvegicus* | AGCAGCAUUGUACAGGGCUAUGA |

**Table S5** List of primers for RT-qPCR

| rCol1a_F | TGACTGGAAGAGCGGAGAGTA |
| --- | --- |
| rCol1a_R | GGGGTTTGGGCTGATGTACC |
| rFN1_F | ATGAGAAGCCTGGATCCCCT |
| rFN1_R | CAGTTGGGGAAGCTCATCTGT |
| rTNC_F | GTGAGGGACGCTCATTCACT |
| rTNC_R | GGGGCAAGGCCTCTGTAATG |
| rACTB_F | CCGCGAGTACAACCTTCTTGC |
| rACTB_R | TATCGTCATCCATGGCGAACTGG |
| rNLRP3_F | CTGCAGAGCCTACAGTTGGG |
| rNLRP3_R | GTCCTGCTTCCACACCTACC |
| rGSDMD_F | CCCTTCCCACAACATCTTCAT |
| rGSDMD_R | TTGGCTTTCCTAGAGGCTGGA |


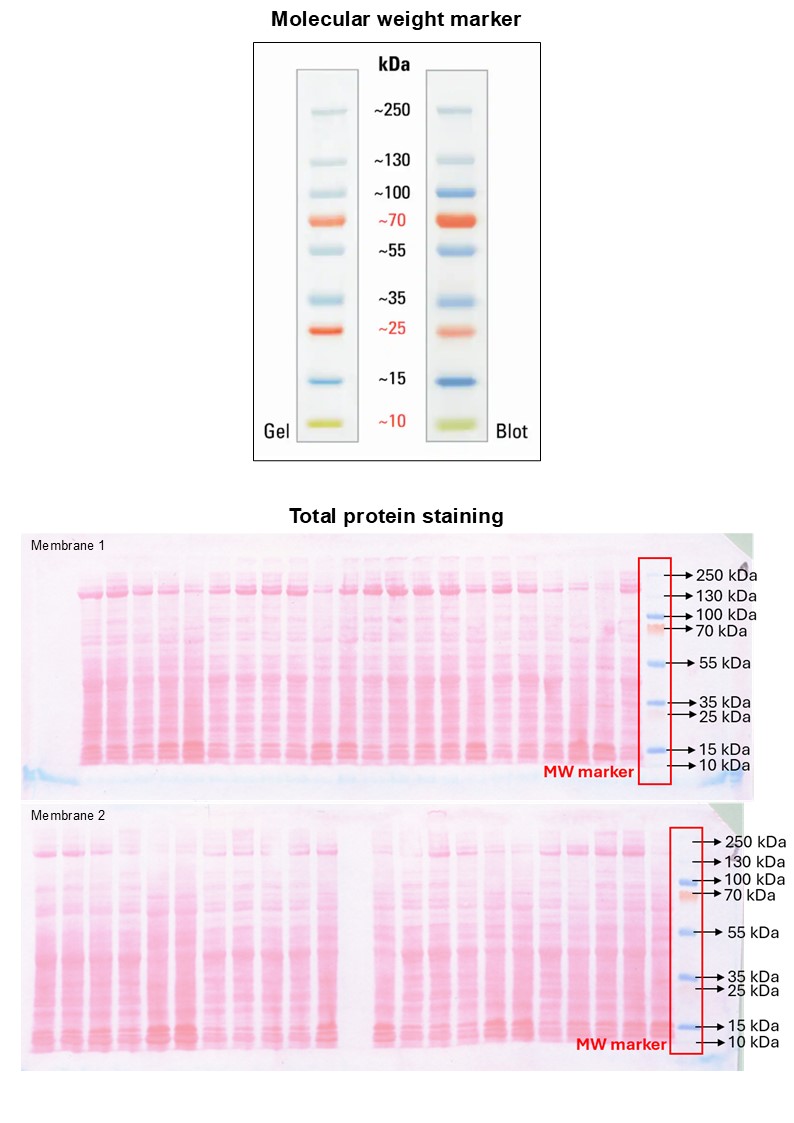
**Supportive material to Western Blot data**


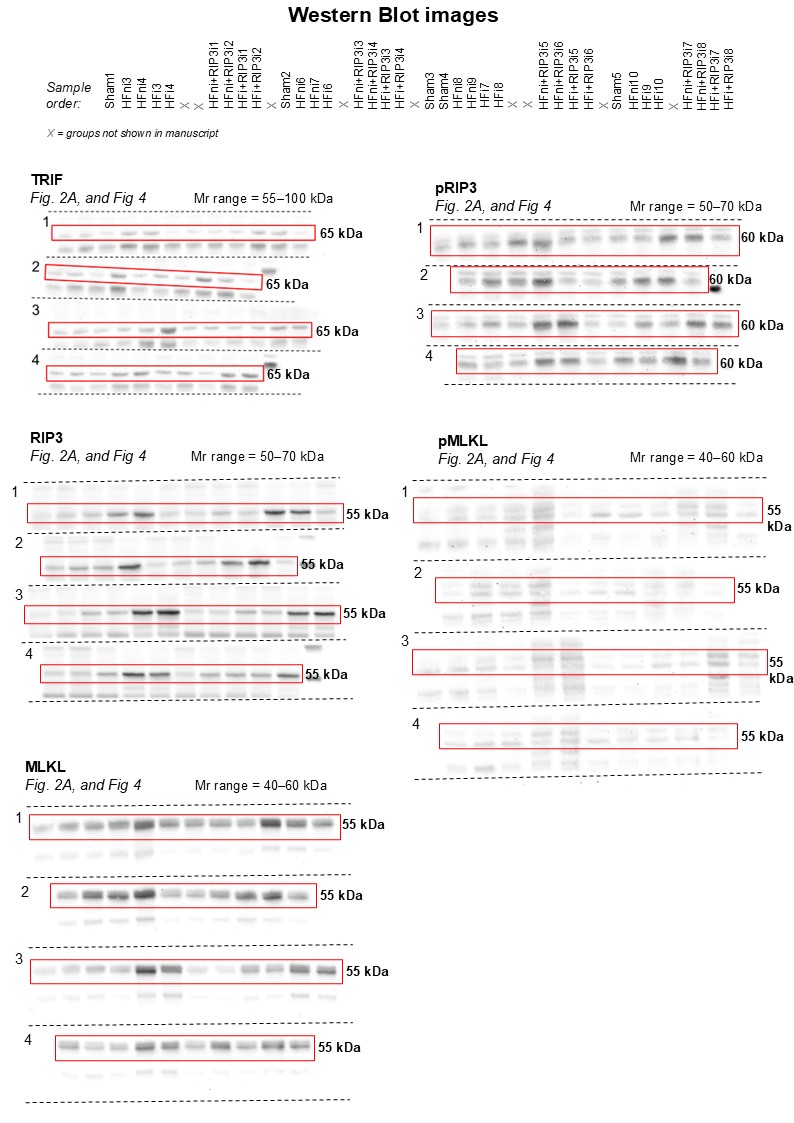


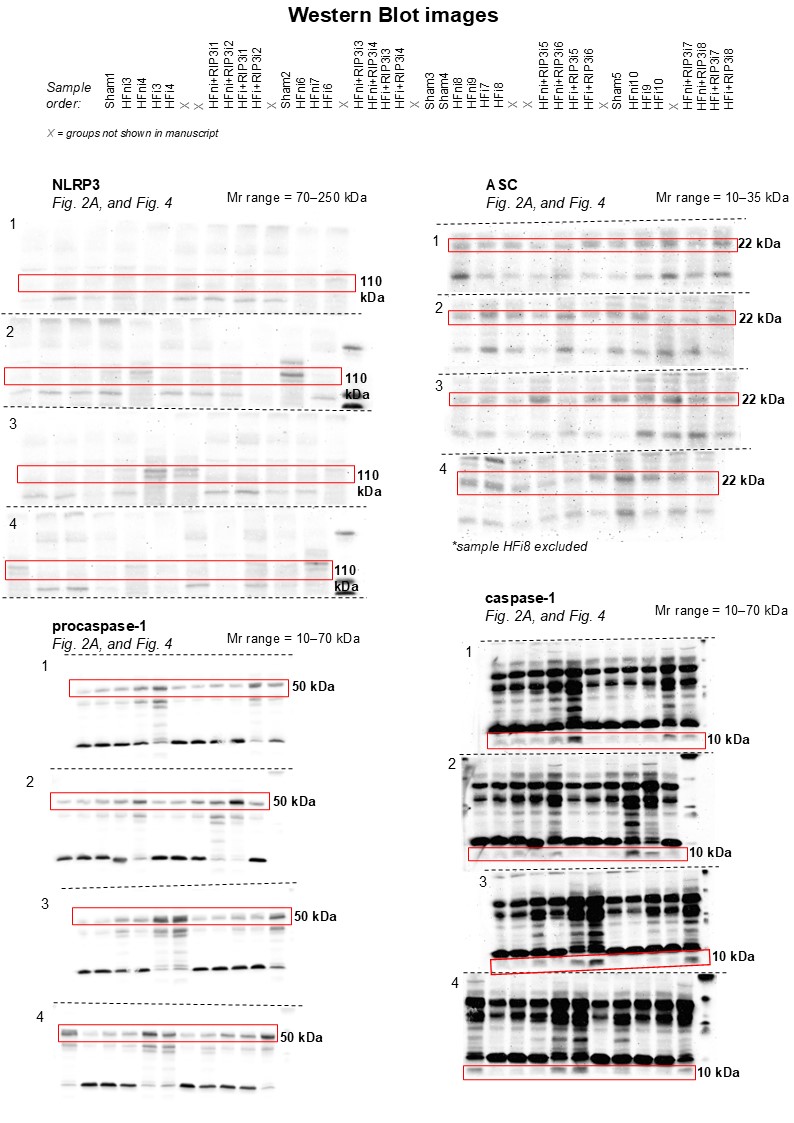


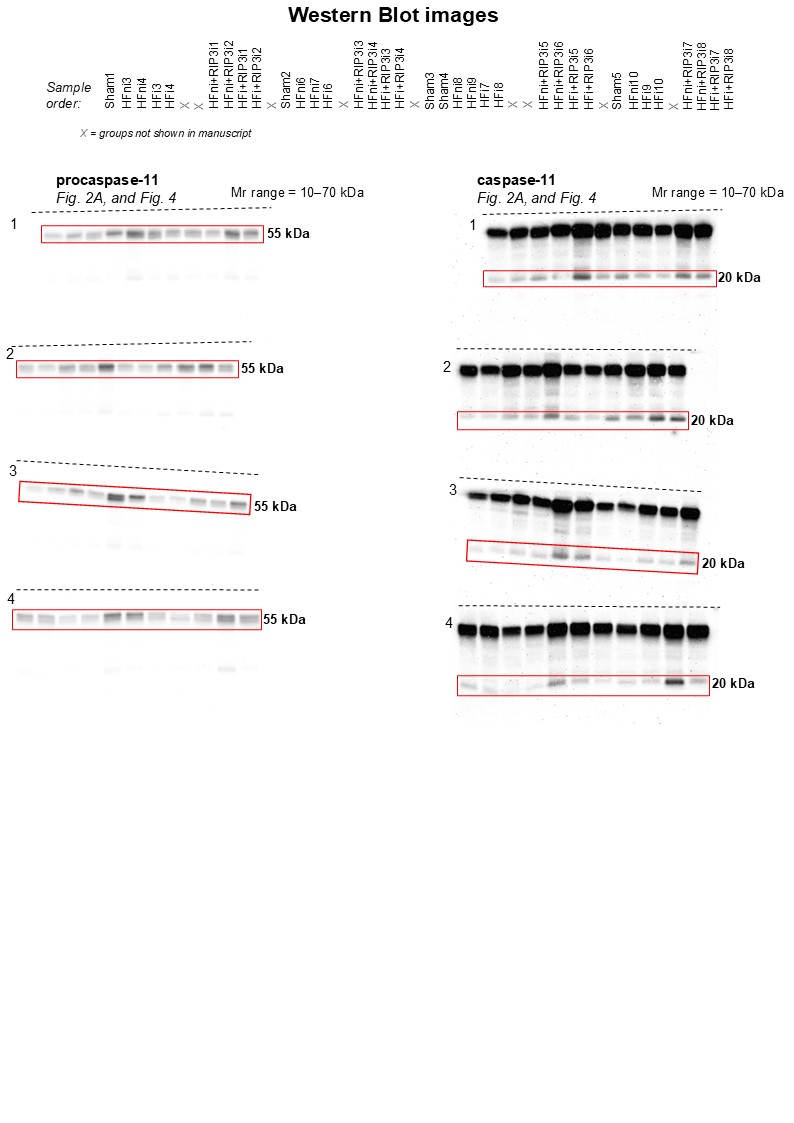


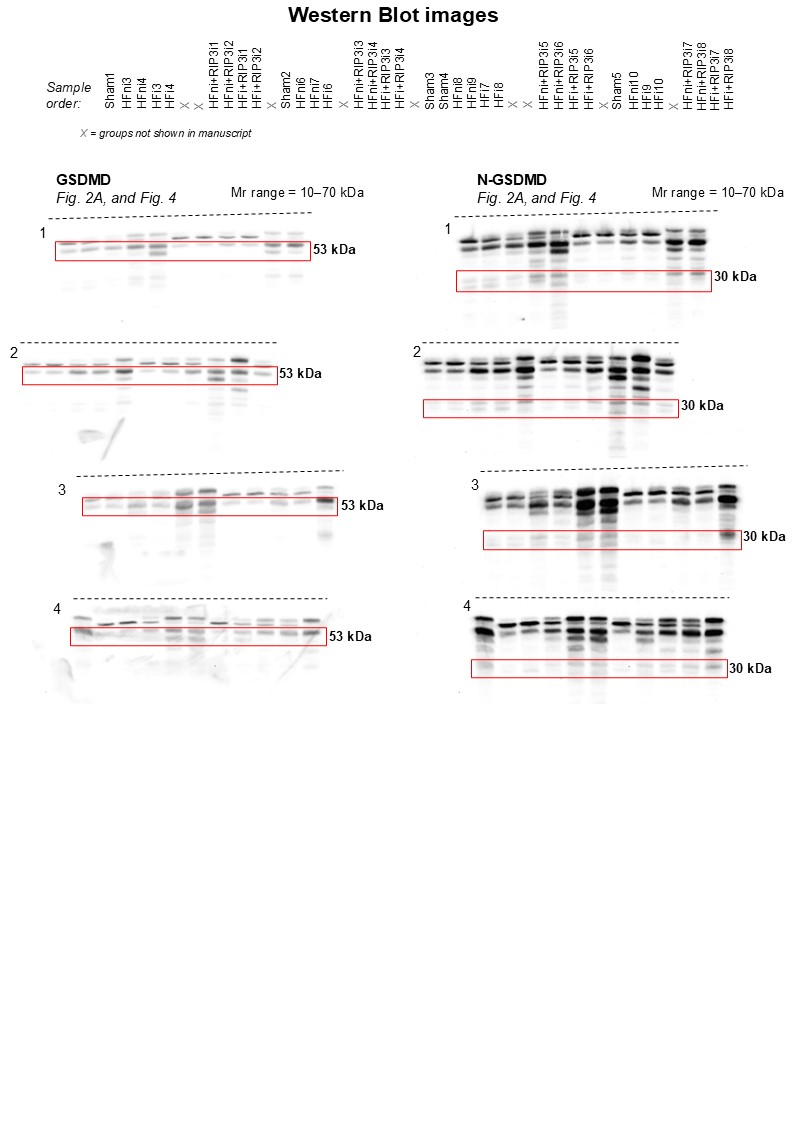


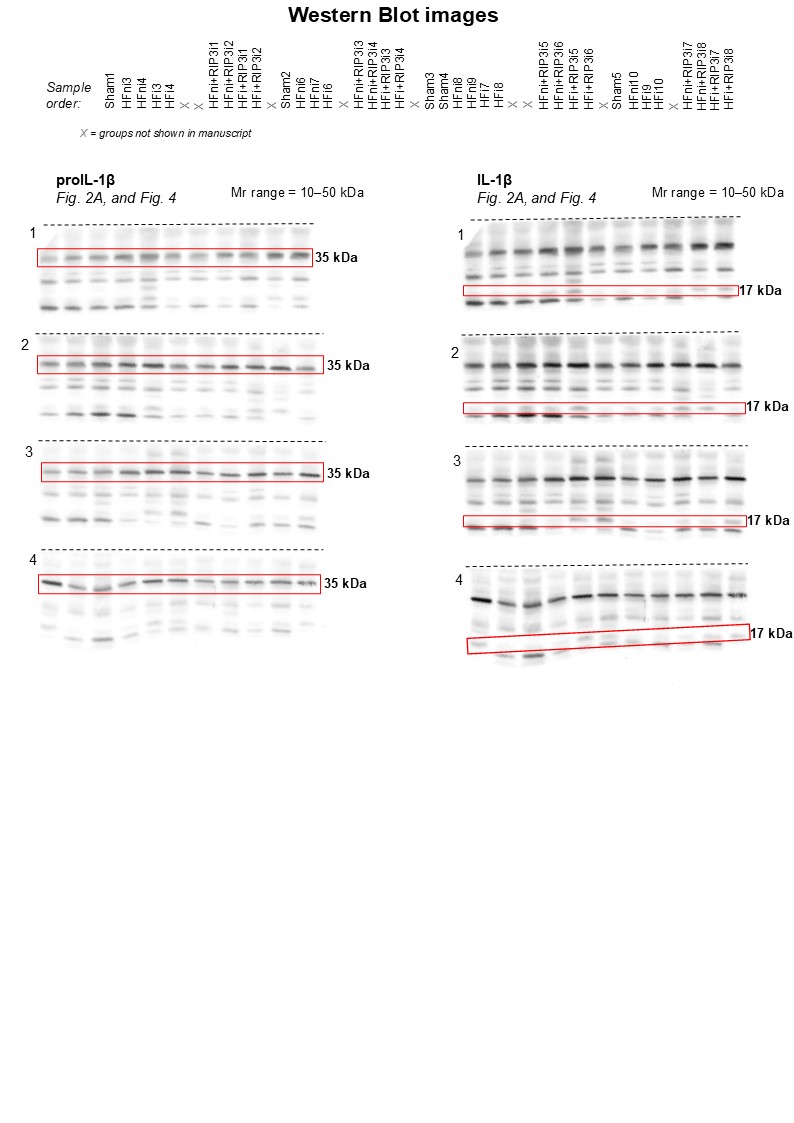


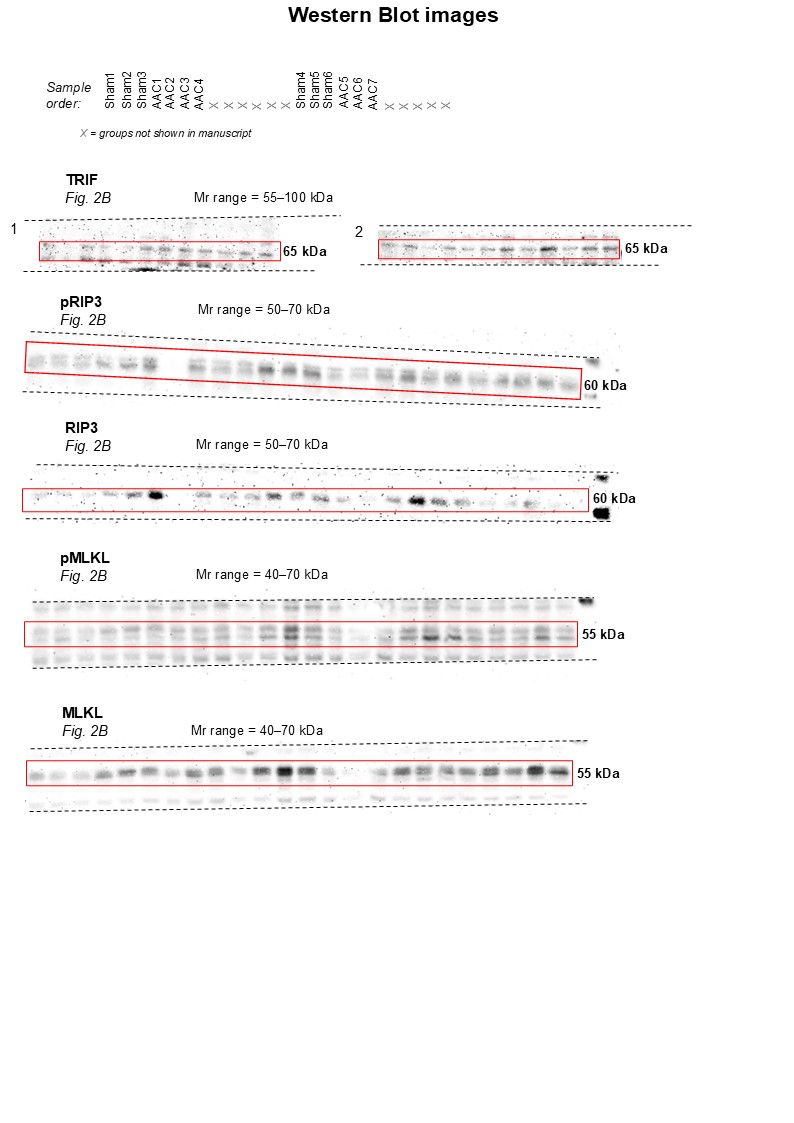


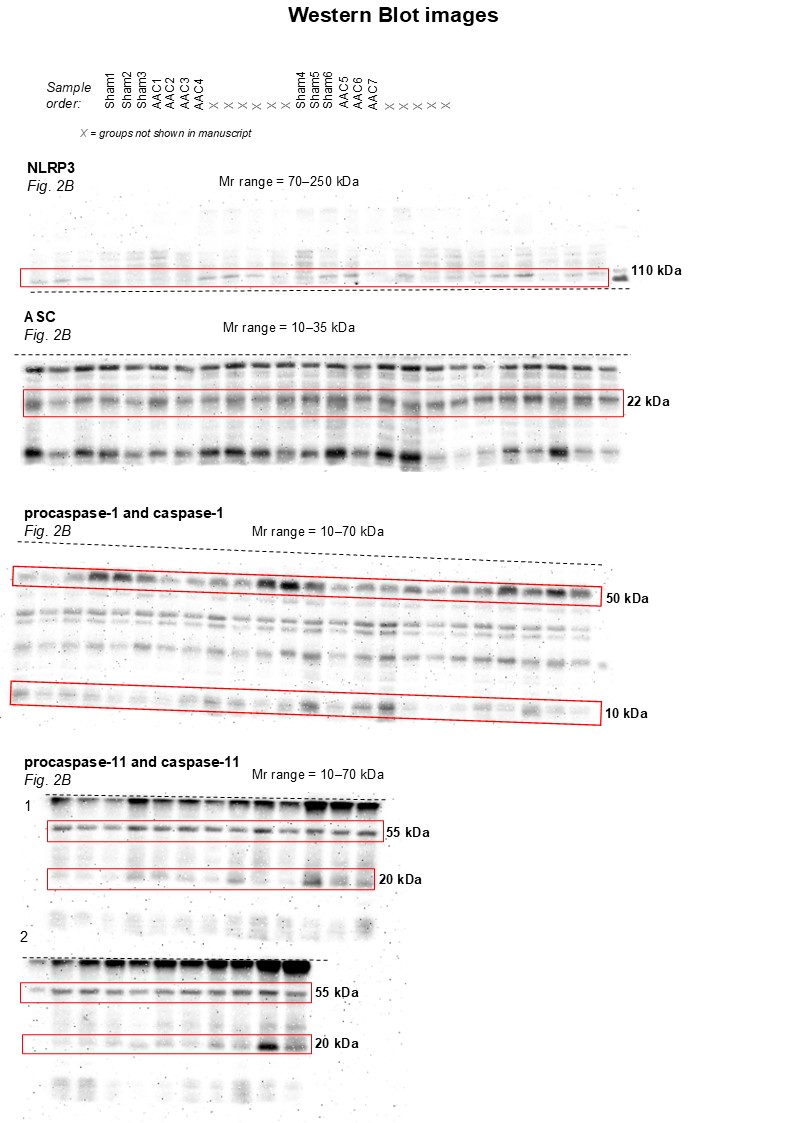


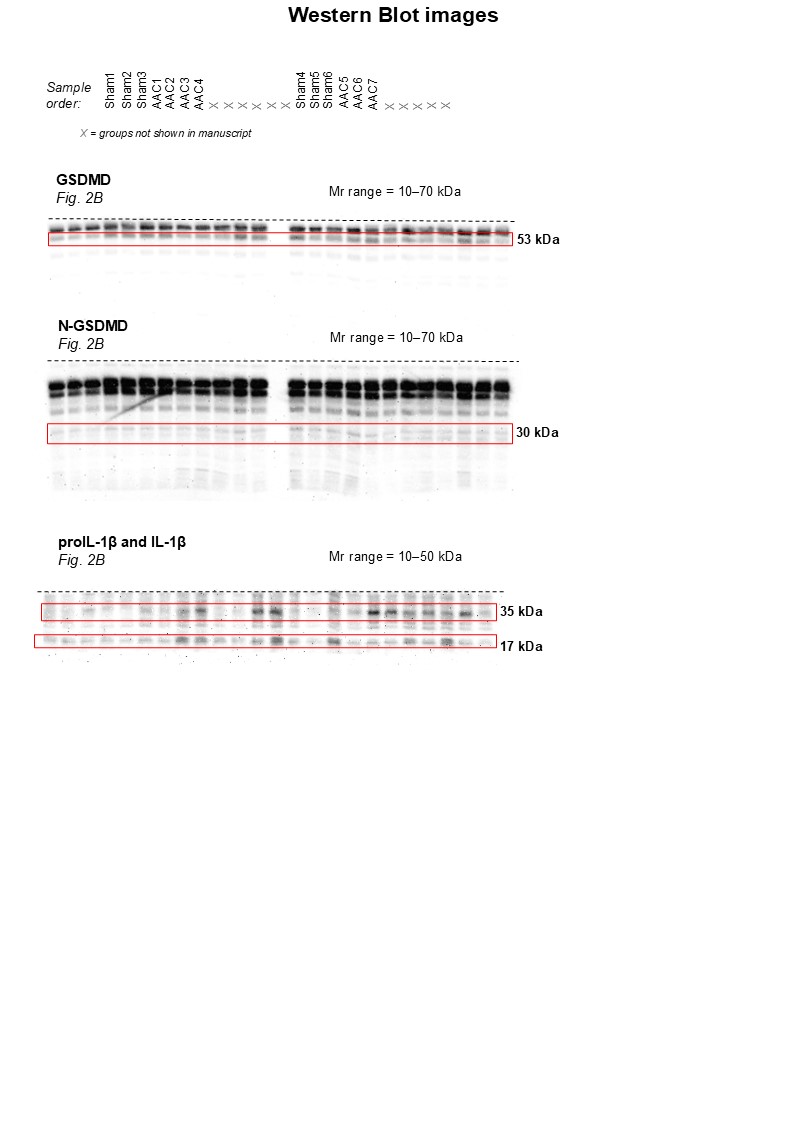


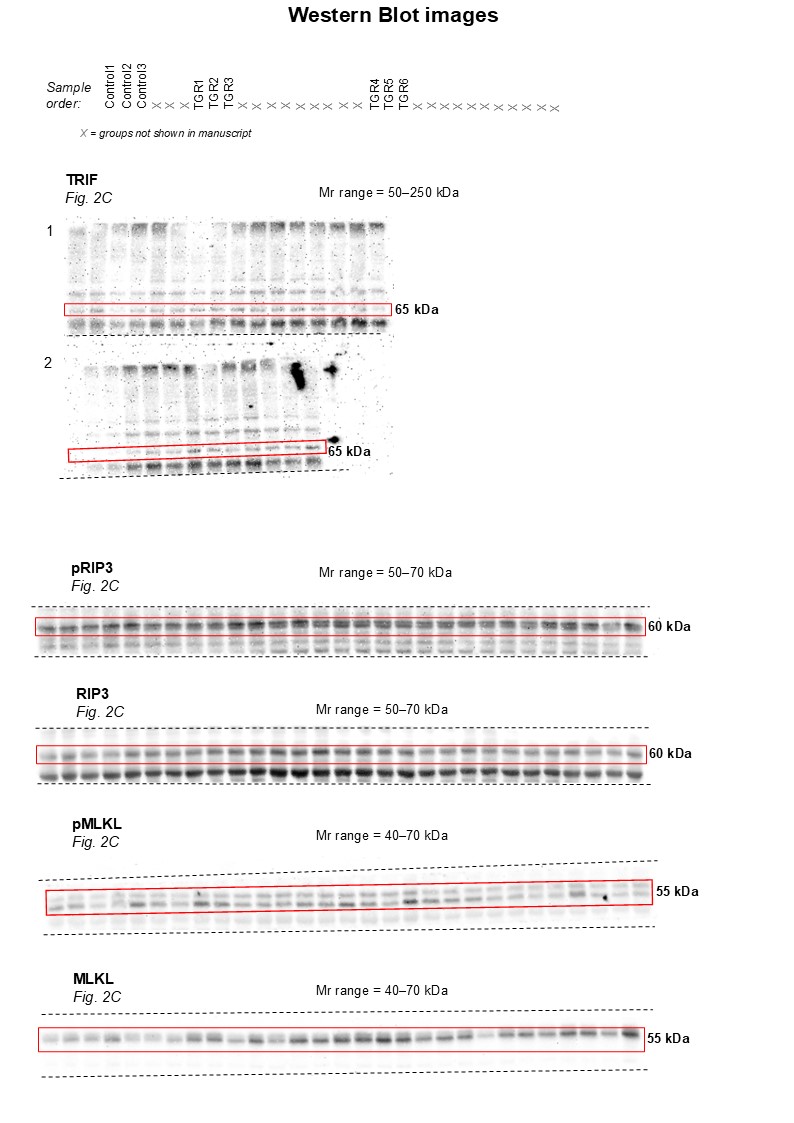


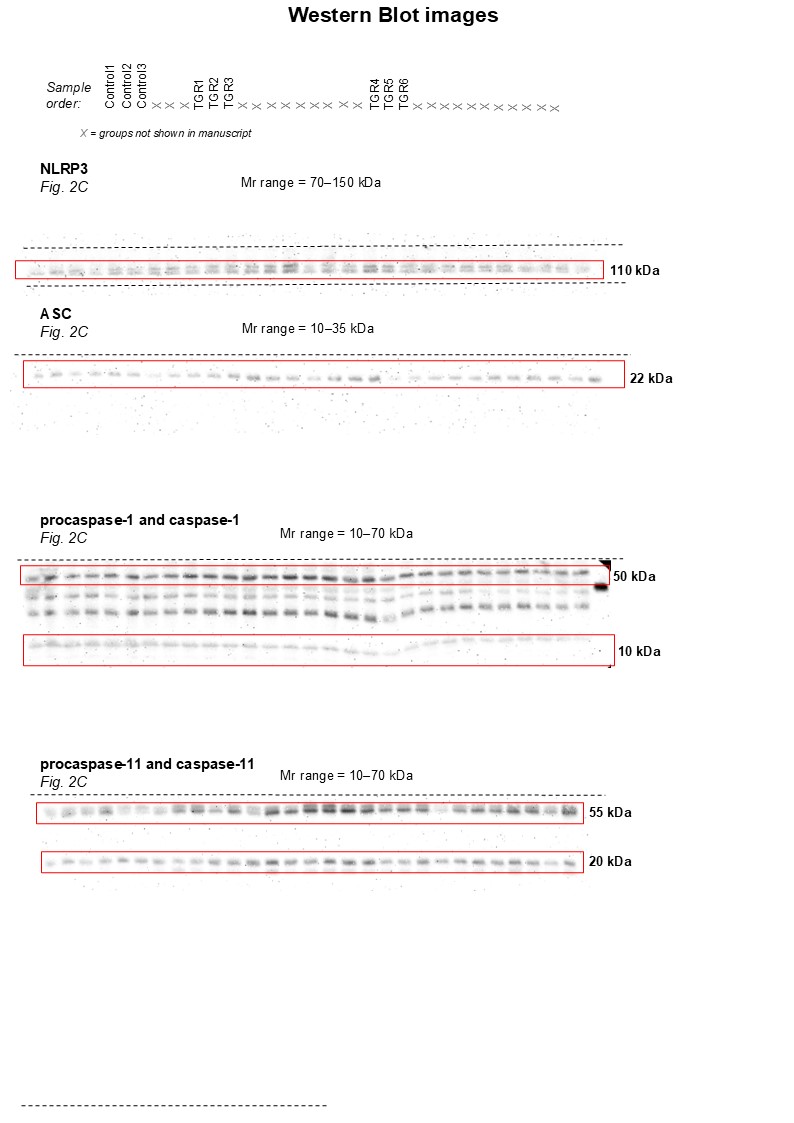


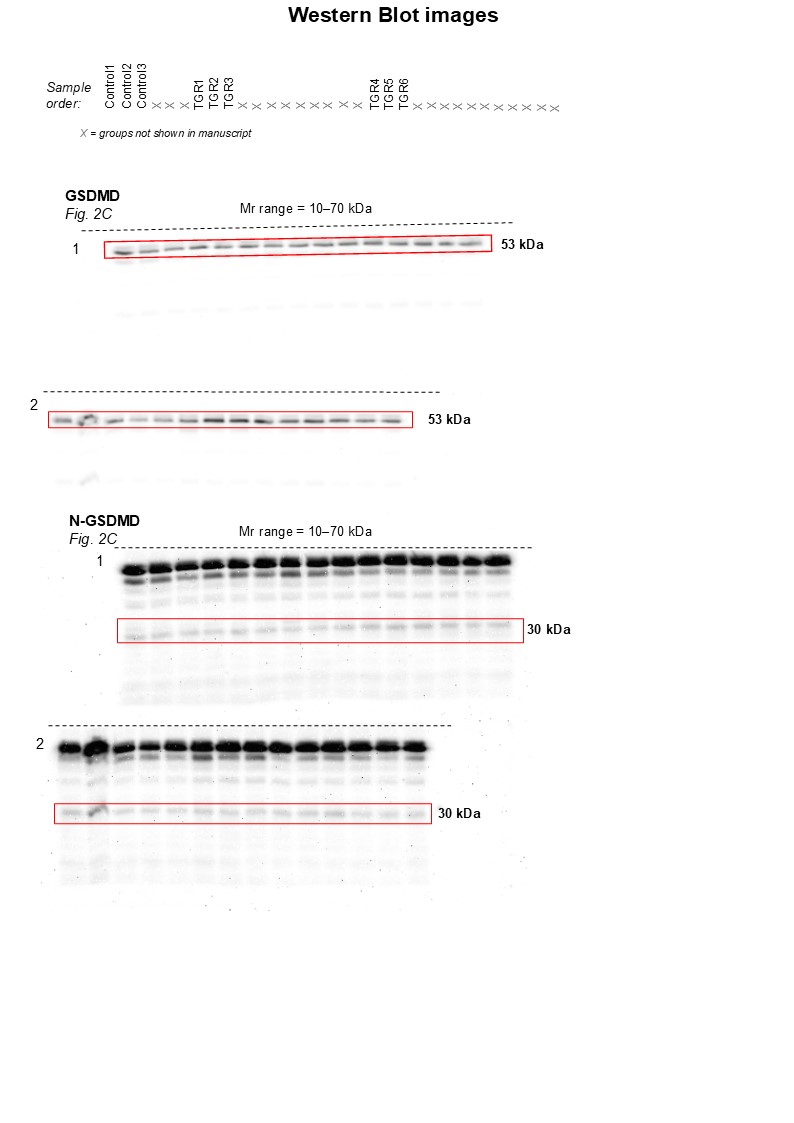


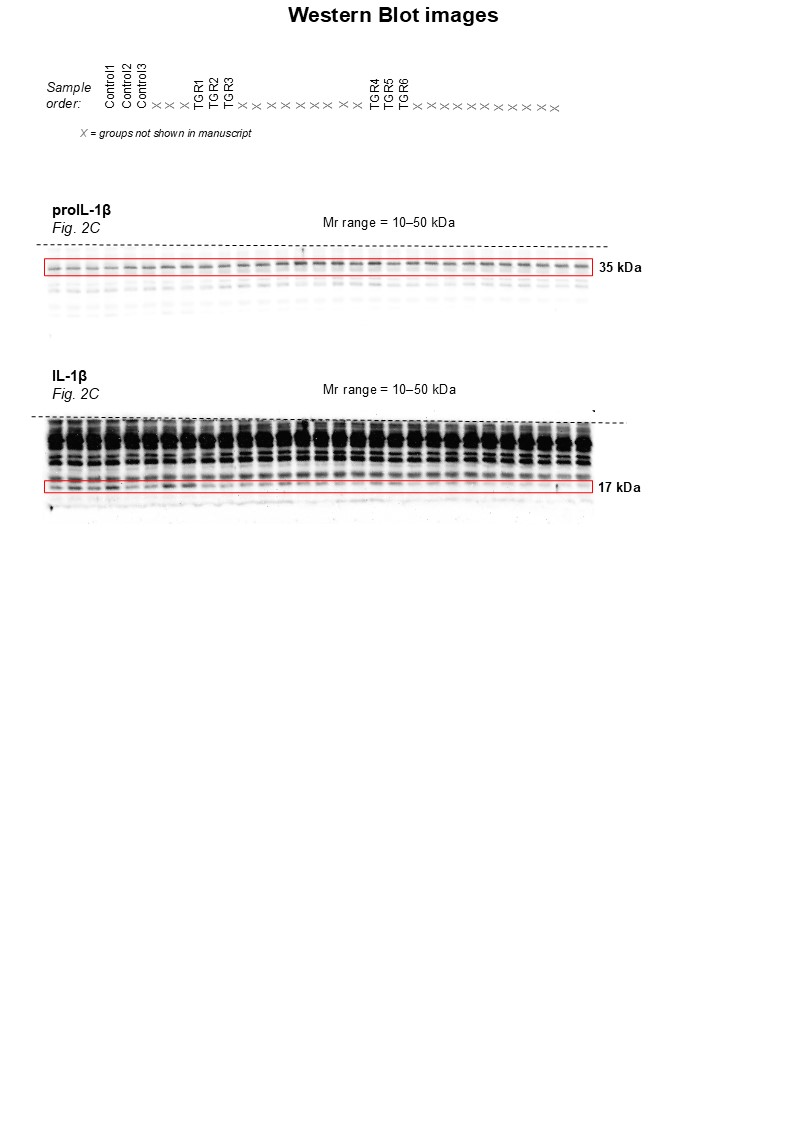


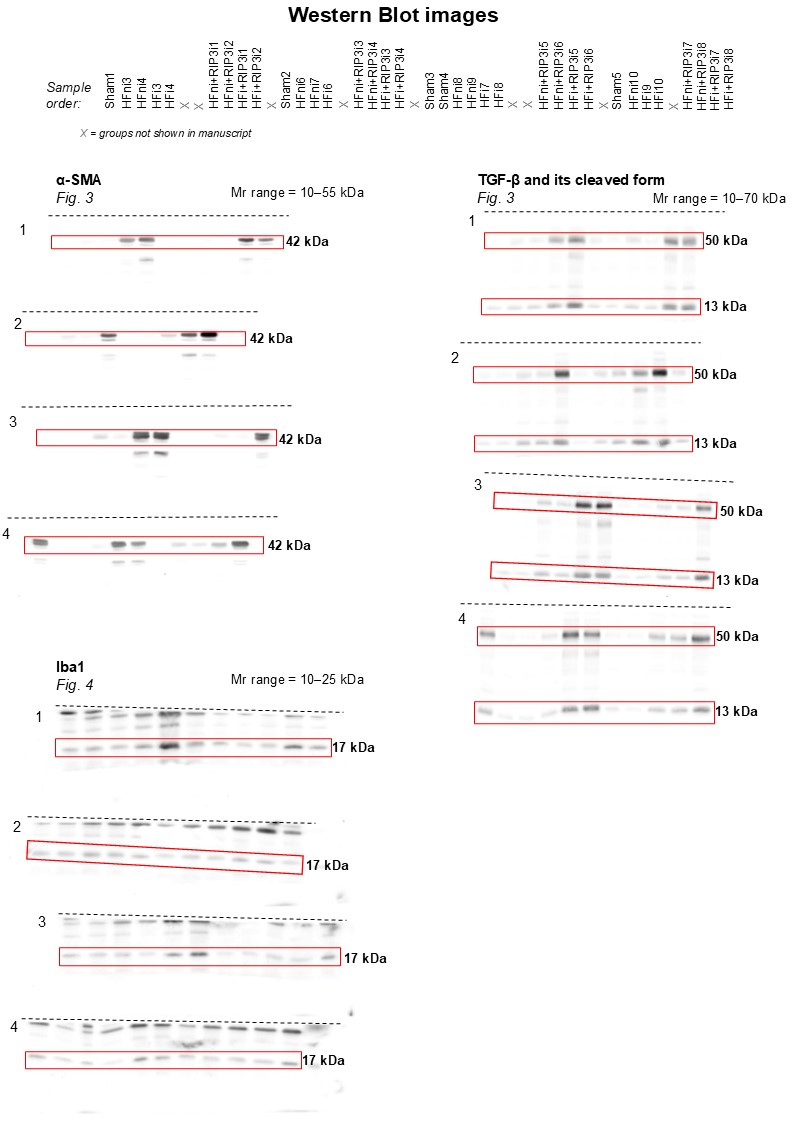


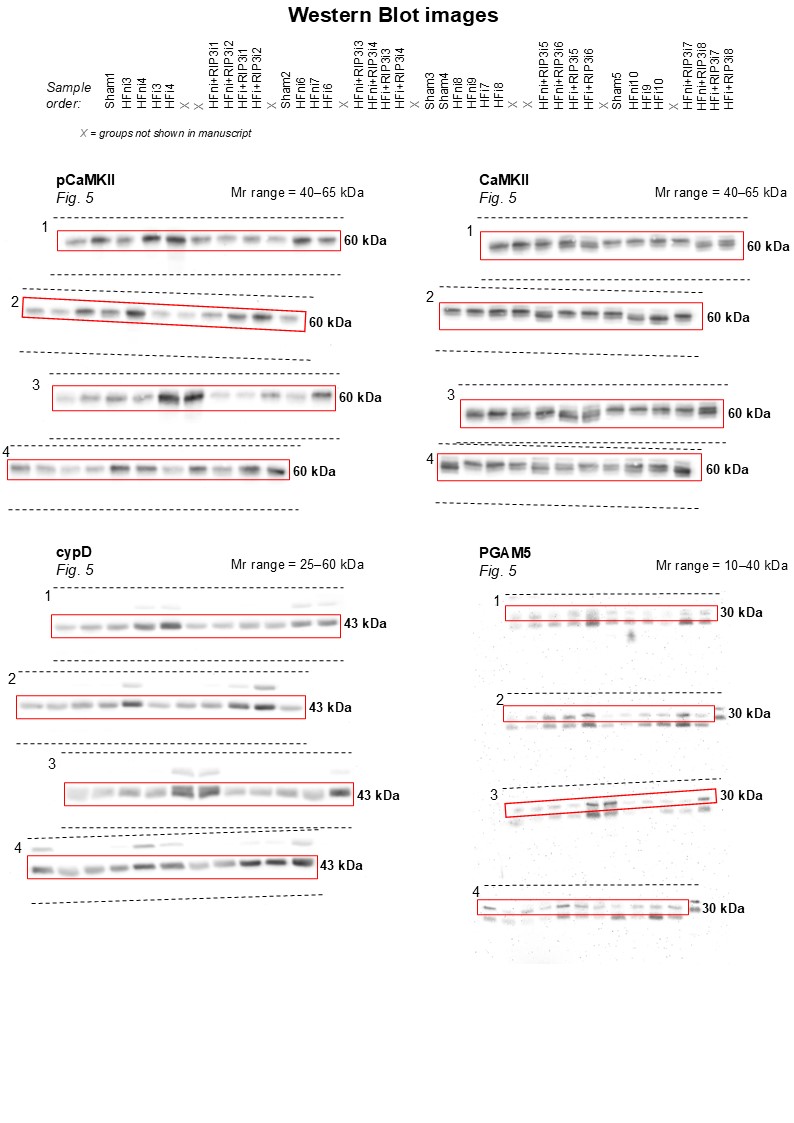


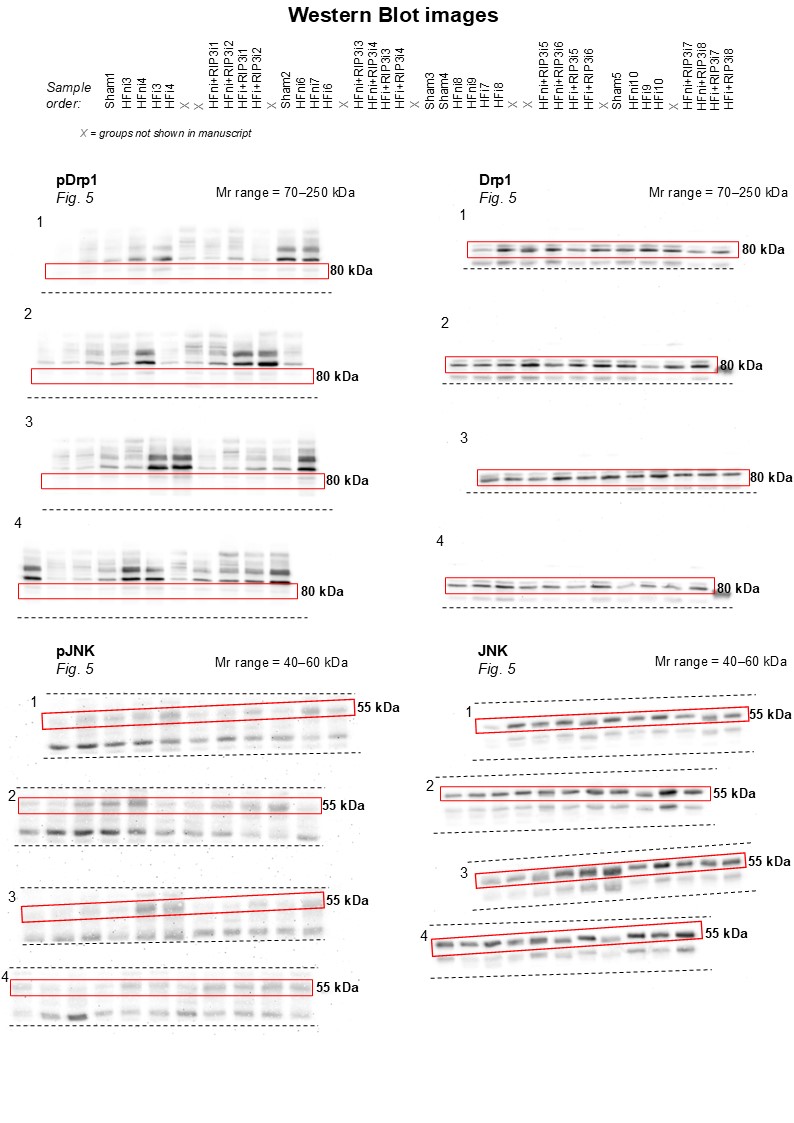


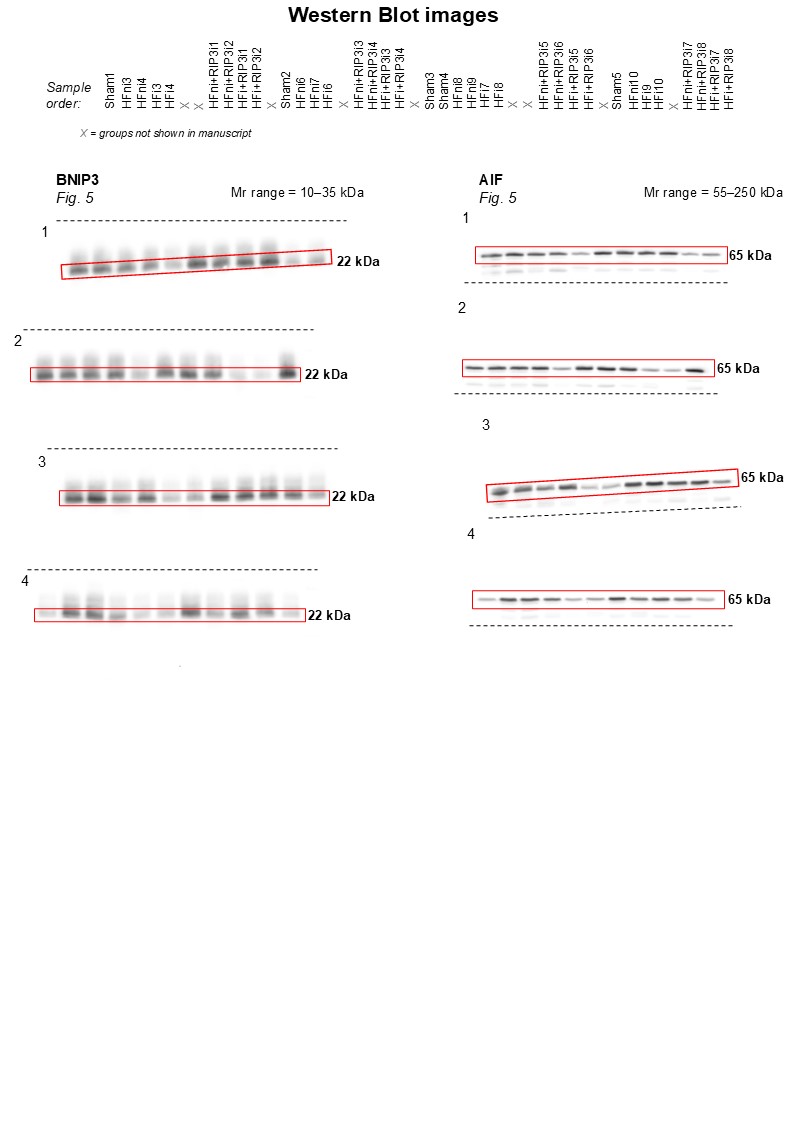


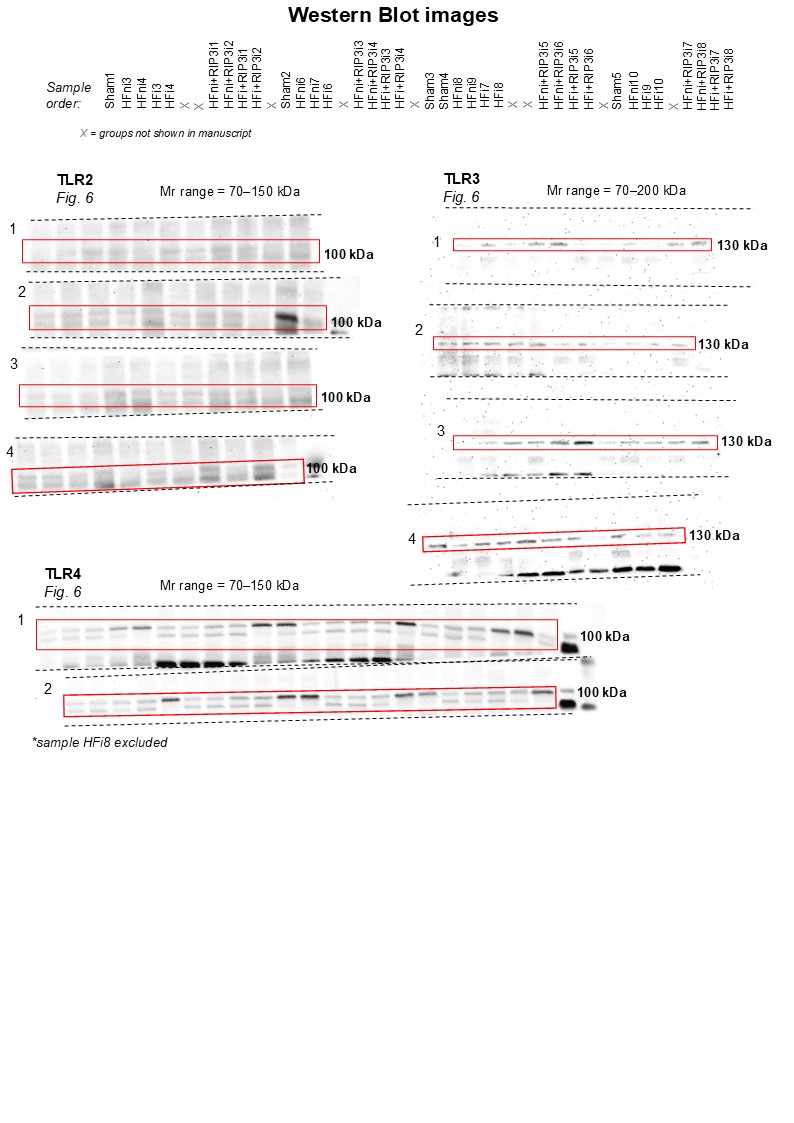


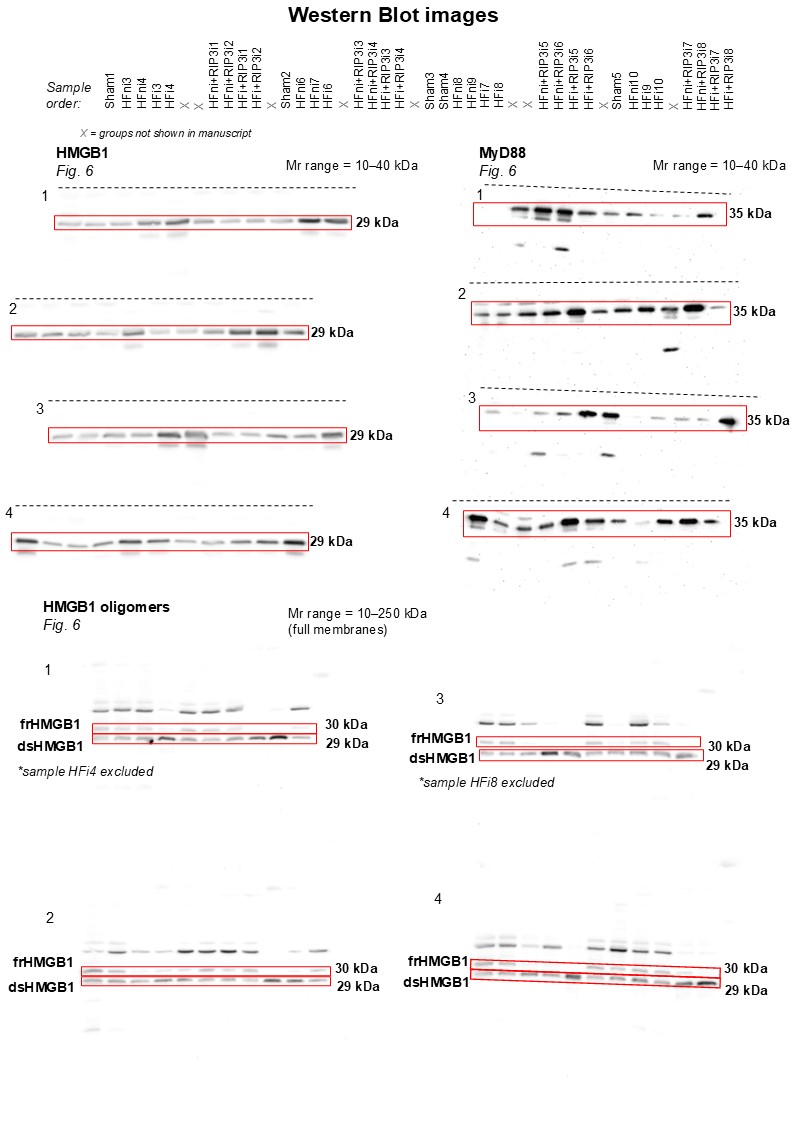


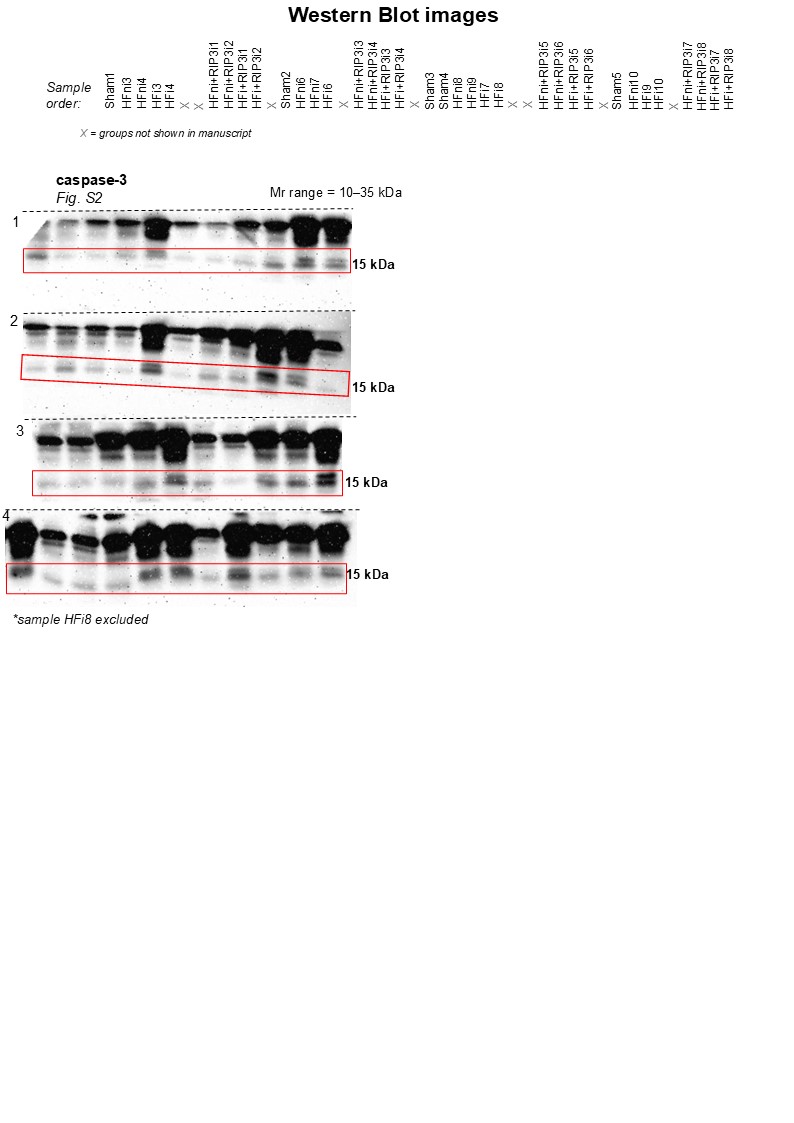


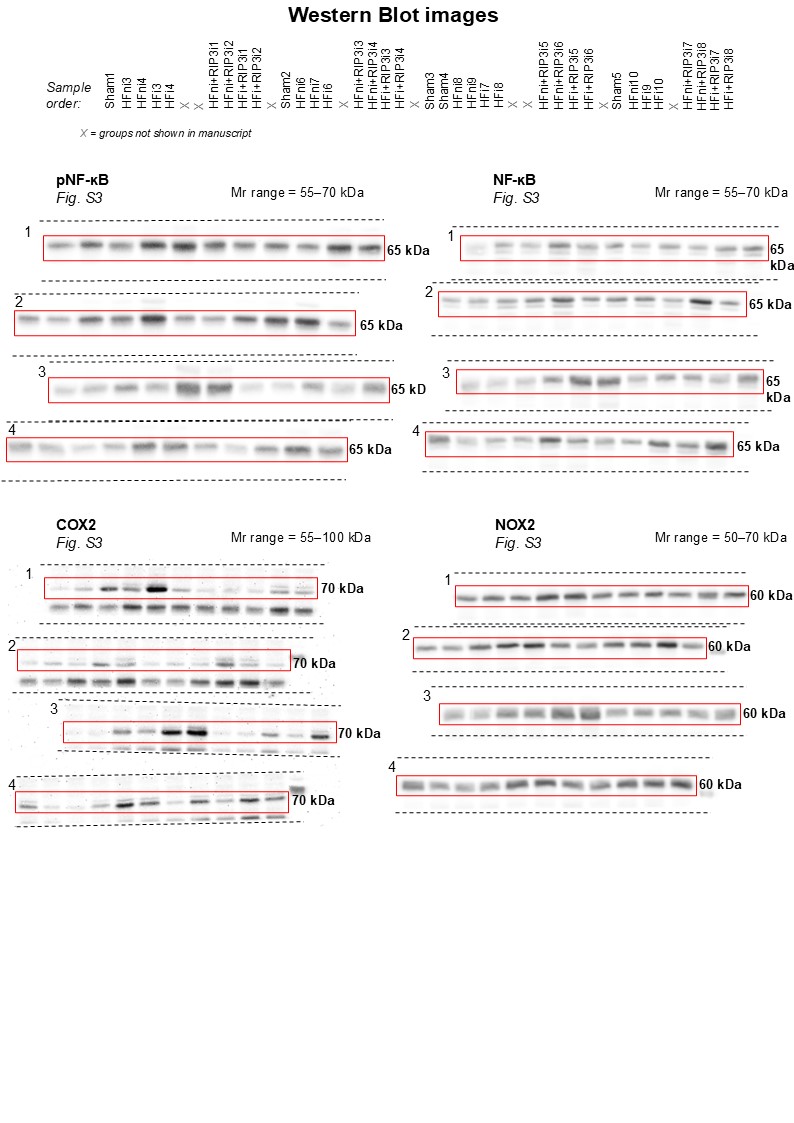


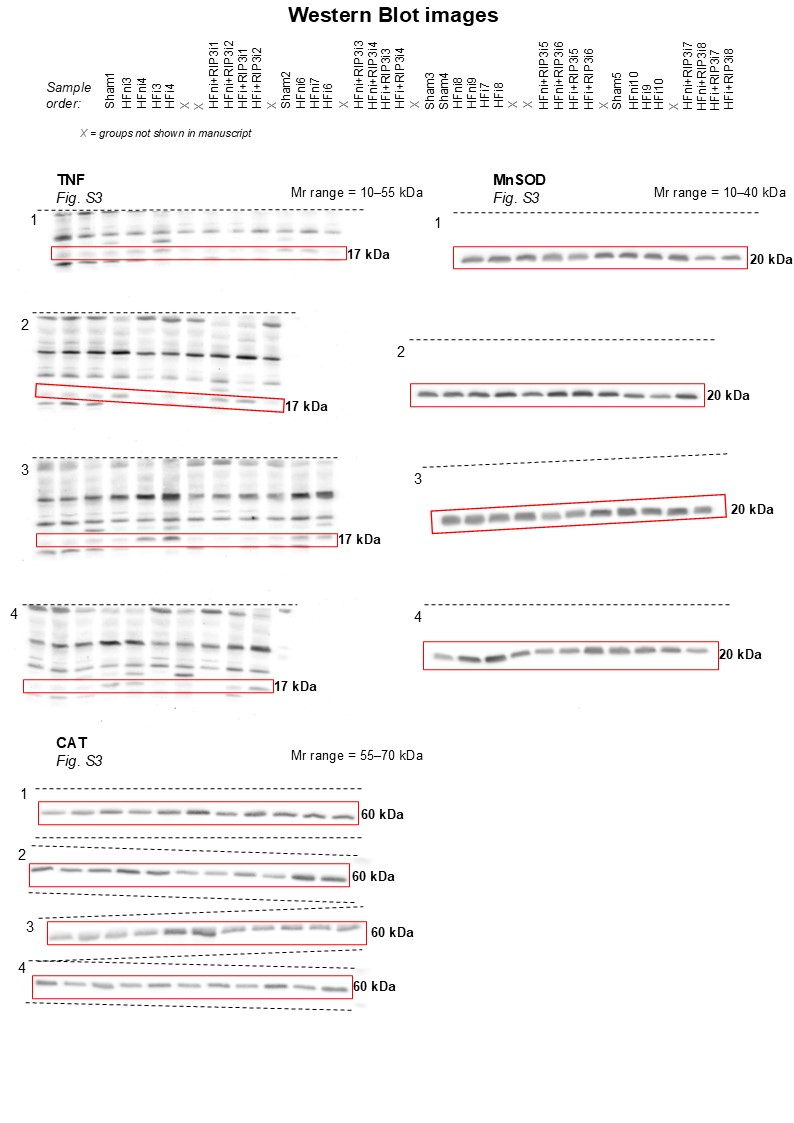


**Summary Fig.** The regulated necrosis-like cell death forms in heart failure due to myocardial infarction or pressure overload, the potential consequences of the plasma membrane rupture and the effect of RIP3 inhibition
