# Supplementary figures and images for: Necrosis-like cell death modes in heart failure: the influence of aetiology and the effects of RIP3 inhibition
Source: Basic Res Cardiol. 2025 Mar 15;120(2):373–92. doi: 10.1007/s00395-025-01101-4 (PMC11976840; doi:10.1007/s00395-025-01101-4)

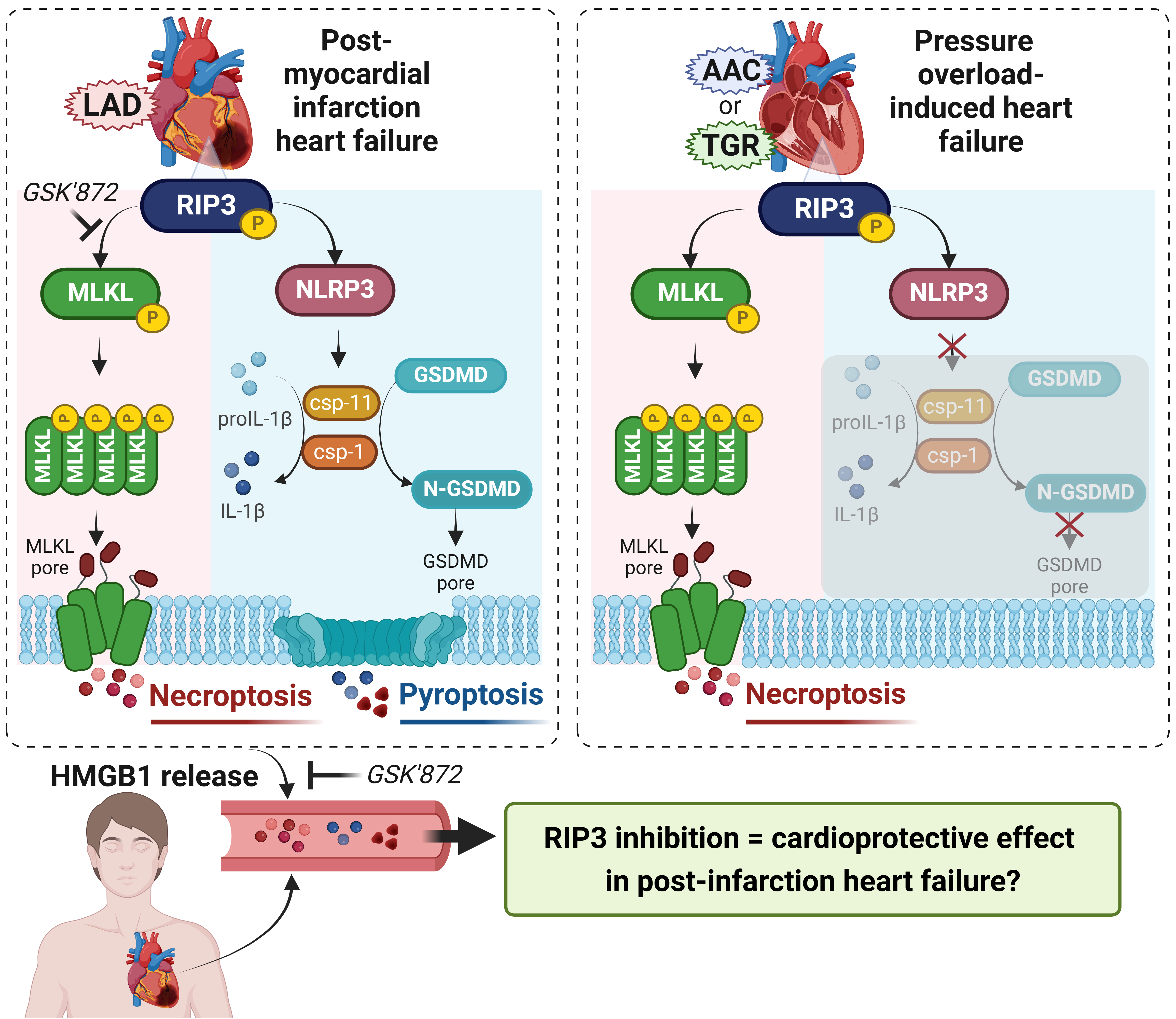

Supplement: Supplementary file 2 — Supplementary file1 (PNG 2012 KB) [file 395_2025_1101_MOESM2_ESM.png]
